# Effect of Planarity of Aromatic Rings Appended to *o*-Carborane on Photophysical Properties: A Series of *o*-Carboranyl Compounds Based on 2-Phenylpyridine- and 2-(Benzo[*b*]thiophen-2-yl)pyridine

**DOI:** 10.3390/molecules24010201

**Published:** 2019-01-07

**Authors:** Hyomin Jin, Seonah Kim, Hye Jin Bae, Ji Hye Lee, Hyonseok Hwang, Myung Hwan Park, Kang Mun Lee

**Affiliations:** 1 Department of Chemistry, Institute for Molecular Science and Fusion Technology, Kangwon National University, Chuncheon 24341, Korea; j_hyomin@naver.com (H.J.); a74747@naver.com (S.K.); jhlee81@kangwon.ac.kr (J.H.L.); hhwang@kangwon.ac.kr (H.H.); 2 Department of Chemistry, KAIST, Daejeon 34142, Korea; qoqoqo9@kaist.ac.kr; 3 Department of Chemistry Education, Chungbuk National University, Cheongju 28644, Korea

**Keywords:** *o*-carborane, intramolecular charge transfer, structural fluctuation, radiative decay

## Abstract

Herein, we investigated the effect of ring planarity by fully characterizing four pyridine-based *o*-carboranyl compounds. *o*-Carborane was introduced to the C4 position of the pyridine rings of 2-phenylpyridine and 2-(benzo[*b*]thiophen-2-yl)pyridine (**CB1** and **CB2**, respectively), and the compounds were subsequently borylated to obtain the corresponding *C*^∧^*N*-chelated compounds **CB1B** and **CB2B**. Single-crystal X-ray diffraction analysis of the molecular structures of **CB2** and **CB2B** confirmed that *o*-carborane is appended to the aryl moiety. In photoluminescence experiments, **CB2**, but not **CB1**, showed an intense emission, assignable to intramolecular charge transfer (ICT) transition between the aryl and *o*-carborane moieties, in both solution and film states. On the other hand, in both solution and film states, **CB1B** and **CB2B** demonstrated a strong emission, originating from π-π * transition in the aryl groups, that tailed off to 650 nm owing to the ICT transition. All intramolecular electronic transitions in these *o*-carboranyl compounds were verified by theoretical calculations. These results distinctly suggest that the planarity of the aryl groups have a decisive effect on the efficiency of the radiative decay due to the ICT transition.

## 1. Introduction

Icosahedral carboranes (C_2_B_10_H_12_) are well-known boron-rich clusters that can be often regarded as 3D analogues of organic aryl derivatives [1]. Among them, organic and organometallic complexes that contain *closo*-*o*-carborane (*closo*-1,2-C_2_B_10_H_12_) have been widely investigated as a novel family of optoelectronic materials for various functional and photonic applications [2,3,4,5,6,7,8,9,10,11,12,13,14,15,16,17,18,19,20,21,22,23] because they definitely possess excellent thermal and electrochemical stabilities. They also realize unique photophysical properties induced by the electron-withdrawing character of the *o*-carborane unit [24,25,26,27,28]. Introduction of this unit to an aryl group leads to the formation of a donor-acceptor conjugated system owing to the strongly electron-withdrawing C atoms and highly delocalized 3D aromaticity of the *o*-carborane cage. Such a good conjugated system results in intramolecular charge transfer (ICT) transition between the two components [29,30,31,32,33,34,35,36,37,38,39,40,41,42,43,44,45,46,47,48,49,50,51]. Thus, the unprecedented luminescent properties of numerous *o*-carboranyl compounds emerge from the ICT-based emissive features [29,30,31,32,33,34,35,36,37,38,39,40,41,42,43,44,45,46,47,48,49,50,51,52,53,54,55,56,57]. For those reasons, *o*-carborane-containing organic and organometallic luminophores have been extensively spotlighted in optoelectronic research fields.

Recently, it has been reported that the intrinsic nature derived from the ICT transition in *o*-carboranyl compounds can be controlled by molecular geometry and structural fluctuation. Fox and co-workers [32] have reported a series of *C*-diazaboryl-*o*-carboranes that exhibit switching of emissive states between a locally excited high-energy state and low-energy ICT state. This phenomenon depends on the dihedral angle between the diazaboryl moiety and C–C bond of *o*-carborane. In addition, various fluorophores possessing *o*-carboranyl groups have exhibited multiple photoluminescence (PL) originating from the alternation of the twisted ICT state [43,44,45,46,47,48,49,50,51]. These results clearly indicate that structural features can play an important role in controlling the intrinsic photophysical and electronic characteristics of *o*-carboranyl compounds.

Thus far, there have been several studies on the unique emission behavior of fluorescent aromatic derivatives conjugated with the C atoms of *o*-carborane, which result from structural variations such as the rotation of appended aromatic groups. However, a detailed investigation of the correlation between such structural variations and the photophysical properties of *o*-carboranyl compounds has been rarely performed. Our group has recently presented for the first time the dramatic change of the emissive ICT transition caused by distortion of biphenyl rings through comparison of the photophysical properties of biphenyl- and fluorene-based *o*-carboranyl compounds [58].

**Figure 1:**
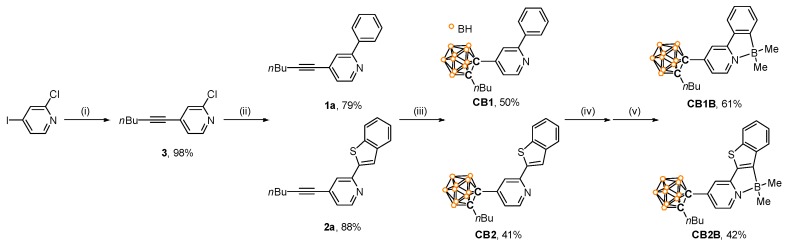
Synthetic procedure for *o*-carboranyl compounds (**CB1**, **CB2**, **CB1B**, and **CB2B**). Reaction conditions: (i) 1-hexyne, CuI, PdCl_2_(PPh_3_)_2_, NEt_3_/toluene, r.t., 24 h. (ii) Pd(PPh_3_)_4_, Na_2_CO_3_, THF/H_2_O (4:1, *v*/*v*), 80 °C, 24 h. (iii) B_10_H_14_, Et_2_S, toluene, 110 °C, 72 h. (iv) BBr_3_, (i-Pr)_2_NEt, DCM, r.t., 48 h. (v) AlMe_3_, toluene, r.t., 0.5 h.

Therefore, to intimately investigate the efect of structural variation in the aromatic group appended to *o*-carborane, we examined the photophysical properties of a series of *o*-carboranyl compounds, namely 2-phenylpyridine (ppy) and 2-(benzo[*b*]thiophen-2-yl)pyridine (btp) with an *o*-carborane substituent at the C4 position of the pyridine ring (**CB1** and **CB2**, respectively, Figure 1), and their corresponding Me_2_B-*C*^∧^*N*-chelated compounds **CB1B** and **CB2B**. The UV-vis and PL experiments were performed to examine how the efficiency of the emissive ICT transition is affected by structural variation, especially the planarity of the aryl groups linked to the *o*-carborane cage. Moreover, the experimental and theoretical structural features and electronic transition states of **CB1**, **CB2**, **CB1B**, and **CB2B** are presented herein in detail.

## 2. Materials and Methods

### 2.1. General Considerations

All operations were carried out under an inert N_2_ atmosphere using standard Schlenk and glove box techniques. Anhydrous-grade toluene and tetrahydrofuran (THF) (Sigma–Aldrich, St. Louis, MO, USA) were dried by passing through an activated alumina column and storing over activated molecular sieves (5 Å). Spectrophotometric-grade toluene was used as received from Alfa Aesar. Na_2_CO_3_, copper(I) iodide (CuI), diethyl sulfide (Et_2_S), tetrakis(triphenylphosphine)palladium(0) (Pd(PPh_3_)_4_), triethylamine, bis(triphenylphosphine)palladium(II) dichloride (Pd(PPh_3_)_2_Cl_2_), boron tribromide (BBr_3_, 1.0 M in dichloromethane), trimethylaluminum (AlMe_3_, 2.0 M in toluene), and poly(methylmethacrylate) (PMMA) were purchased from Sigma–Aldrich; *n*-hexane, dichloromethane, methanol, acetone, and ethyl acetate, from Alfa Aesar; benzo[*b*]thien-2-yl-boronic acid, phenylboronic acid, 2-chloro-4-iodopyridine, 1-hexyne, trimethylamine, and *N*,*N*-diisopropylamine, from TCI Chemicals; and decaborane (B_10_H_14_), from KatChem. These commercially available reagents and solvents were used without further purification. Deuterated solvents were purchased from Cambridge Isotope Laboratories and used after drying over activated molecular sieves (5 Å). The NMR spectra of all compounds were recorded at ambient temperature on the Bruker Avance 400 spectrometer (400.13, 100.62, and 128.38 MHz for ^1^H, ^13^C, and ^11^B, respectively). Chemical shifts are given in ppm and referenced against either external Me_4_Si (^1^H and ^13^C) or BF_3_·Et_2_O (^11^B). Elemental analyses were performed on the EA3000 elemental analyzer (Eurovector, at the Central Laboratory of Kangwon National University, Gangwon-do, Korea). The UV-vis absorption and PL spectra were recorded on the Jasco V-530 (JASCO International Co., Ltd., Tokyo, Japan) and Horiba FluoroMax-4P spectrophotometers (Horiba FluoroMax*^®^*, Salem, NH, USA), respectively. Fluorescence decay lifetimes (*τ*) were measured at 298 K using a time-correlated single-photon counting spectrometer (FLS920, Edinburgh Instruments, at the Central Laboratory of Kangwon National University) equipped with an EPL-375 ps pulsed semiconductor diode laser as the excitation source and microchannel plate photomultiplier tube (200‒850 nm) as the detector. Absolute PL quantum yields (*Φ*_em_) were obtained at 298 K using an absolute PL quantum yield spectrophotometer (FM-SPHERE, 3.2-inch internal integrating sphere in FluoroMax-4P).

### 2.2. Synthesis of 2-Chloro-4-(hex-1-yn-1-yl)pyridine (**3**)

Toluene (7 mL) and triethylamine (1:9, *v*/*v*) were added using a cannula to a mixture of 2-chloro-4-iodopyridine (1.80 g, 7.5 mmol), CuI (100 mg), and Pd(PPh_3_)_2_Cl_2_ (289 mg) at room temperature. After stirring the resulting dark brown slurry for 15 min, 1-hexyne (1.29 mL, 11.3 mmol) was added. The reaction mixture was then refluxed at 80 °C for 24 h. The volatiles were removed using a rotary evaporator to afford a dark-gray residue. The crude product was purified by silica column chromatography (eluent:dichloromethane/*n*-hexane = 1:2) to yield **3** as an ivory solid (1.43 g, Yield = 98%). ^1^H NMR (CDCl_3_): δ 8.28 (d, *J* = 5.1 Hz, 1H), 7.28 (s, 1H), 7.15 (dd, *J* = 5.1, 1.3 Hz, 1H), 2.43 (t, *J* = 7.0 Hz, 2H), 1.59 (m, 2H), 1.47 (m, 2H), 0.95 (t, *J* = 7.3 Hz, 3H). ^13^C NMR (CDCl_3_): δ 151.60, 149.38, 135.28, 126.24, 124.46, 97.75 (acetylene-*C*), 77.44 (acetylene-*C*), 30.33, 22.02, 19.18, 13.56. Anal. Calcd for C_11_H_12_ClN: C, 68.22; H, 6.25; N, 7.23. Found: C, 68.01; H, 6.11; N, 7.14.

### 2.3. Synthesis of ***1a***

To a mixture of **3** (0.77 g, 4 mmol) and Pd(PPh_3_)_4_ (0.60 g, 0.52 mmol) in THF (30 mL) was successively added phenylboronic acid (0.58 g, 4.8 mmol) and Na_2_CO_3_ (1.27 g, 12.0 mmol) in H_2_O (10 mL). The mixture was stirred and refluxed at 80 °C for 24 h. After cooling it to room temperature, 30 mL of water was added. The organic portions were dried over MgSO_4_ and filtered. Following evaporation of the solvent under reduced pressure, the yellow residue was purified by column chromatography (eluent: dichloromethane/*n*-hexane = 1:3, *v*/*v*) to yield **1a** as a pale yellow oil (0.74 g, Yield = 79%). ^1^H NMR (CDCl_3_): δ 8.58 (d, *J* = 5.0 Hz, 1H), 7.96 (d, *J* = 8.0 Hz, 2H), 7.69 (s, 1H), 7.42 (m, 3H), 7.17 (d, *J* = 5.0 Hz, 1H), 2.44 (t, *J* = 7.1 Hz, 2H), 1.61 (m, 2H), 1.49 (m, 2H), 0.95 (t, *J* = 7.3 Hz, 3H). ^13^C NMR (CDCl_3_): δ 157.44, 149.49, 138.96, 133.03, 129.09, 128.72, 126.90, 124.07, 122.75, 95.65 (acetylene-*C*), 78.74 (acetylene-*C*), 30.50, 22.04, 19.19, 13.60. Anal. Calcd for C_17_H_17_N: C, 86.77; H, 7.28; N, 5.95. Found: C, 86.44; H, 7.07; N, 5.60.

### 2.4. Synthesis of ***2a***

A procedure analogous to that for **1a** using **3** (0.58 g, 3.0 mmol), benzo[*b*]thiophene-2-ylboronic acid (0.64 g, 3.6 mmol), Pd(PPh_3_)_4_ (0.35 g, 0.30 mmol), and Na_2_CO_3_ (0.95 g, 9.0 mmol) was employed to afford **2a** as a white solid (0.77 g, Yield = 88%). ^1^H NMR (CDCl_3_): δ 8.52 (d, *J* = 5.1 Hz, 1H), 7.85 (t, *J* = 4.6 Hz, 1H), 7.81 (s, 1H), 7.78 (t, *J* = 4.6 Hz, 1H), 7.75 (s, 1H), 7.34 (m, 2H), 7.14 (dd, *J* = 5.1, 1.4 Hz, 1H), 2.45 (t, *J* = 7.1 Hz, 2H), 1.62 (m, 2H), 1.49 (m, 2H), 0.96 (t, *J* = 7.3 Hz, 3H). ^13^C NMR (DMSO): δ 152.56, 149.46, 144.28, 140.67, 140.36, 132.97, 125.06, 124.48, 124.11, 122.54, 121.66, 121.30, 96.21 (acetylene-*C*), 78.38 (acetylene-*C*), 30.43, 22.02, 19.18, 13.58. Anal. Calcd for C_19_H_7_NS: C, 78.31; H, 5.88; N, 4.81. Found: C, 78.12; H, 5.70; N, 4.65.

### 2.5. Synthesis of **CB1**

To a toluene solution (100 mL) of B_10_H_14_ (0.46 g, 3.77 mmol) and **1a** (0.68 g, 2.90 mmol) was slowly added an excess amount of Et_2_S (2.5 equiv. for B_10_H_14_) at room temperature. The reaction mixture was further stirred at 110 °C for 3 d. After cooling it to room temperature, the solvent was removed under vacuum, and then methanol (50 mL) was added. The precipitated yellow solid was filtered and re-dissolved in toluene. The solution was purified by passing through an alumina column, and the solvent was removed in vacuo to afford **CB1** as a white solid. Recrystallization from an acetone/methanol mixture gave 0.51 g of **CB1** (Yield = 50%). ^1^H NMR (CDCl_3_): δ 8.72 (d, *J* = 5.3 Hz, 1H), 7.98 (d, *J* = 6.8 Hz, 2H), 7.91 (d, *J* = 0.8 Hz, 1H), 7.49 (m, 3H), 7.43 (dd, *J* = 5.3, 1.8 Hz, 1H), 3.70‒1.61 (br, 10H, CB-B*H*), 1.79 (t, *J* = 8.6 Hz, 2H), 1.38 (m, 2H), 1.09 (m, 2H), 0.73 (t, *J* = 7.3 Hz, 3H). ^13^C NMR (CDCl_3_): δ 158.69, 150.45, 140.06, 138.09, 129.90, 129.04, 127.09, 123.28, 122.08, 82.35 (CB-*C*), 80.75 (CB-*C*), 35.08, 31.63, 22.10, 13.52. ^11^B NMR (CDCl_3_): δ −3.73 (br s, 1B), −4.57 (br s, 1B), −10.83 (br s, 8B). Anal. Calcd for C_17_H_27_B_10_N: C, 57.76; H, 7.70; N, 3.96. Found: C, 57.70; H, 7.55; N, 3.72.

### 2.6. Synthesis of **CB2**

A procedure analogous to that for **CB1** using B_10_H_14_ (0.42 g, 3.45 mmol), **2a** (0.77 g, 2.65 mmol), and Et_2_S (2.5 equiv. for B_10_H_14_) was employed. Recrystallization from an acetone/methanol mixture afforded **CB2** as a yellow solid (0.45 g, Yield = 41%). ^1^H NMR (CDCl_3_): δ 8.65 (d, *J* = 5.3 Hz, 1H), 7.97 (s, 1H), 7.90 (s, 1H), 7.86 (m, 2H), 7.38 (m, 3H), 3.56‒1.68 (br, 10H, CB-B*H*), 1.81 (t, *J* = 8.0 Hz, 2H), 1.40 (m, 2H), 1.10 (m, 2H), 0.74 (t, *J* = 7.3 Hz, 3H). ^13^C NMR (CDCl_3_): 153.81, 150.50, 143.28, 140.93, 140.21, 140.05, 125.69, 124.85, 124.45, 123.56, 122.65, 122.36, 120.94, 82.40 (CB-*C*), 80.40 (CB-*C*), 35.10, 31.64, 22.10, 13.51. ^11^B NMR (CDCl_3_): δ −3.65 (br s, 1B), −4.47 (br s, 1B), −10.82 (br s, 8B). Anal. Calcd for C_19_H_27_B_10_NS: C, 55.72; H, 6.64; N, 3.42. Found: C, 55.50; H, 6.44; N, 3.22.

### 2.7. Synthesis of **CB1B**

To a stirred solution of **CB1** (0.41 g, 1.17 mmol) and diisopropylamine (0.20 mL, 1.17 mmol) in dichloromethane (2.0 mL) at 0 °C was added BBr_3_ (1.0 M in dichloromethane, 3.5 mL, 3.5 mmol). After stirring the reaction mixture at room temperature for 48 h, saturated K_2_CO_3_ aqueous solution was added. The organic layer was separated, and the aqueous layer was extracted with dichloromethane (3 × 10 mL). The combined organic portions were dried over MgSO_4_ and filtered. Evaporation of the solvent under reduced pressure afforded a crude solid residue. Recrystallization from a dichloromethane/*n*-hexane mixture afforded the BBr_2_-*C*^∧^*N*-chelated compound of **CB1** as a white solid (0.50 g, Yield = 81%). This compound was characterized by ^1^H NMR spectra only and then used in the subsequent methylation reaction in situ. ^1^H NMR (CDCl_3_): δ 8.93 (d, *J* = 6.3 Hz, 1H), 8.05 (d, *J* = 1.5 Hz, 1H), 7.87 (d, *J* = 7.4 Hz, 1H), 7.82 (d, *J* = 7.7 Hz, 1H), 7.72 (dd, *J* = 6.3, 1.9 Hz, 1H), 7.61 (t, *J* = 7.4 Hz, 1H), 7.46 (t, *J* = 7.6 Hz, 1H), 3.44‒1.75 (br, 10H, CB-B*H*), 1.86 (t, *J* = 8.5 Hz, 2H), 1.45 (m, 2H), 1.17 (m, 2H), 0.78 (t, *J* = 7.3 Hz, 3H).

To a stirred solution of the BBr_2_-*C*^∧^*N*-chelated compound of **CB1** (0.30 g, 0.57 mmol) in toluene (5.0 mL) at room temperature was added AlMe_3_ (2.0 M in toluene, 0.63 mL, 1.26 mmol). After stirring the mixture at room temperature for 30 min, the reaction was quenched by adding distilled water (7 mL). The organic layer was separated, and the aqueous layer was extracted with ethyl acetate (3 × 10 mL). The combined organic portions were dried over MgSO_4_ and filtered. Evaporation of the solvent under reduced pressure afforded **CB1B**. Recrystallization from a dichloromethane/*n*-hexane mixture afforded **CB1B** as a white solid (0.14 g, Yield = 61%). ^1^H NMR (CDCl_3_): δ 8.45 (d, *J* = 6.1 Hz, 1H), 8.11 (s, 1H), 7.89 (d, *J* = 7.7 Hz, 1H), 7.65 (d, *J* = 7.2 Hz, 1H), 7.53 (dd, *J* = 6.0, 1.7 Hz, 1H), 7.47 (t, *J* = 7.2 Hz, 1H), 7.33 (t, *J* = 7.5 Hz, 1H), 3.46‒1.66 (br, 10H, CB-B*H*), 1.84 (t, *J* = 8.5 Hz, 2H), 1.42 (m, 2H), 1.13 (m, 2H), 0.74 (t, *J* = 6.3 Hz, 3H), 0.05 (s, 6H, B(C*H*_3_)_2_). ^13^C NMR (CDCl_3_): δ 157.84, 142.82, 142.70, 134.03, 131.30, 129.47, 125.56, 122.69, 121.93, 119.65, 82.80 (CB-*C*), 79.52 (CB-*C*), 35.23, 31.70, 22.06, 13.50, 8.83 (B(*C*H_3_)_2_). ^11^B NMR (CDCl_3_): δ 0.53 (*B*(CH_3_)_2_, 1B), −3.19 (br s, 1B), −4.48 (br s, 1B), −10.71 (br s, 8B). Anal. Calcd for C_19_H_32_B_11_N: C, 58.01; H, 8.20; N, 3.56. Found: C, 57.92; H, 8.10; N, 3.34.

### 2.8. Synthesis of **CB2B**

A procedure analogous to that for the BBr_2_-*C*^∧^*N*-chelated compound of **CB1** using **CB2** (0.45 g, 1.09 mmol), diisopropylamine (0.20 mL, 1.39 mmol), and BBr_3_ (1.0 M in dichloromethane, 3.27 mL, 3.27 mmol) was employed. Recrystallization from a dichloromethane/*n*-hexane mixture afforded the BBr_2_-*C*^∧^*N*-chelated compound of **CB2** as a yellow solid (0.57 g, Yield = 90%). This compound was characterized by ^1^H NMR spectra only and then used in the subsequent methylation reaction in situ. ^1^H NMR (CDCl_3_): δ 8.86 (d, *J* = 6.3 Hz, 1H), 8.21 (dd, *J* = 6.8, 1.8 Hz, 1H), 7.92 (dd, *J* = 6.9, 1.5 Hz, 1H), 7.68 (d, *J* = 1.5 Hz, 1H), 7.59 (dd, *J* = 6.3, 1.8 Hz, 1H), 7.50 (m, 1H), 3.47‒1.68 (br, 10H, CB-B*H*), 1.89 (t, *J* = 8.52 Hz, 2H), 1.45 (m, 2H), 1.19 (m, 2H), 0.79 (t, *J* = 7.3 Hz, 3H).

A procedure analogous to that for **CB1B** using the BBr_2_-*C*^∧^*N*-chelated compound of **CB2** (0.30 g, 0.52 mmol) and AlMe_3_ (2.0 M in toluene, 0.55 mL, 1.1 mmol) was employed. Recrystallization from a dichloromethane/*n*-hexane mixture afforded **CB2B** as a pale-yellow solid (0.10 g, Yield = 43%). ^1^H NMR (CDCl_3_): δ 8.39 (d, *J* = 6.1 Hz, 1H), 8.00 (m, 1H), 7.92 (dd, *J* = 6.3, 2.8 Hz, 1H), 7.71 (d, *J* = 1.5 Hz, 1H), 7.41 (m, 3H), 3.57‒1.74 (br, 10H, CB-B*H*), 1.86 (t, *J* = 8.54 Hz, 2H), 1.43 (m, 2H), 1.15 (m, 2H), 0.75 (t, *J* = 7.3 Hz, 3H), 0.15 (s, 6H, B(C*H*_3_)_2_). ^13^C NMR (CDCl_3_): δ 154.49, 146.38, 143.03, 142.96, 140.26, 131.99, 126.68, 126.46, 124.71, 123.47, 120.60, 119.44, 82.82 (CB-*C*), 79.40 (CB-*C*), 35.21, 31.72, 22.07, 13.52, 8.13 (B(*C*H_3_)_2_). ^11^B NMR (CDCl_3_): δ 0.14 (*B*(CH_3_)_2_, 1B), −3.19 (br s, 1B), −4.47 (br s, 1B), −10.56 (br s, 8B). Anal. Calcd for C_19_H_32_B_11_N: C, 55.72; H, 6.64; N, 3.42. Found: C, 55.62; H, 6.42; N, 3.25.

### 2.9. UV-vis Absorption and PL Measurements

Solution UV-vis absorption and PL measurements for the *o*-carboranyl compounds were performed in degassed toluene (5.0 × 10^−5^ M) at 298 K using a 1-cm quartz cuvette. The PL measurements were also carried out in toluene solution at 77 K and film (5 wt % doped on PMMA) on 1.5 × 1.5 cm quartz plates (thickness = 1 mm) at 298 K. The absolute PL quantum yields (*Φ*_em_) at the solution and film states were obtained at 298 K using an absolute PL quantum yield spectrophotometer (FM-SPHERE, 3.2-inch internal integrating sphere on FluoroMax-4P).

### 2.10. X-ray Crystallography

Single crystals of **CB2** and **CB2B** suitable for X-ray diffraction were grown from a dichloromethane/*n*-hexane mixture. The single crystals were coated with Paratone oil and mounted onto glass capillaries. Crystallographic measurements were performed on the Bruker D8 QUEST CCD area detector diffractometer with a graphite-monochromated Mo-Kα radiation (*λ* = 0.71073 Å). The structures were solved by direct methods and all non-hydrogen atoms were subjected to anisotropic refinement using the full-matrix least-squares method on *F*^2^ on the SHELXTL/PC package to obtain the X-ray crystallographic data in CIF format (CCDC 1878407 and 1878406 for **CB2** and **CB2B**, respectively). Hydrogen atoms on the carbon and boron atoms were placed at their geometrically calculated positions and refined as riding on the corresponding carbon atoms with isotropic thermal parameters. Detailed crystallographic data are given in Tables S1 and S2 in the Supplementary Material.

### 2.11. Theoretical Calculations

The ground (*S*_0_) and first excited state (*S*_1_) structures of the *o*-carboranyl compounds, **CB1**, **CB1B**, **CB2**, and **CB2B** were optimized using density functional theory (DFT) with the B3LYP functional. The 6-31G(d) basis set was used for all atoms [59]. The electronic transition energies were calculated using time-dependent DFT (TD-DFT) [60] based on the hybrid B3LYP functional (TD-B3LYP), which also accounts for electron correlation. All calculations were performed using the GAUSSIAN 09 program [61]. The percent contribution of a functional group to each molecular orbital was calculated using the GaussSum 3.0 program [62].

## 3. Results and Discussion

### 3.1. Synthesis and Characterization

The synthetic pathways for all compounds with an *o*-carboranyl unit appended at the C4 position of the pyridine ring (**CB1**, **CB1B**, **CB2**, and **CB2B**) are illustrated in Figure 1. The Sonogashira reaction between 1-hexyne and 2-chloro-4-iodopyridine produces **3** in high yield (98%). After the Suzuki–Miyaura coupling reaction between **3** and phenylboronic acid (for **1a**) or benzo[*b*]thien-2-yl-boronic acid (for **2a**), **CB1** and **CB2** are synthesized via cage formation with decaborane (B_10_H_14_) in the presence of Et_2_S (Scheme 1) [58,63,64,65]. The *C*^∧^*N*-chelated compounds **CB1B** and **CB2B** were finally obtained in moderate yield (61% and 42%, respectively) via simple borylation of **CB1** and **CB2**, respectively, with BBr_3_, followed by treatment with AlMe_3_.

**Figure 2:**
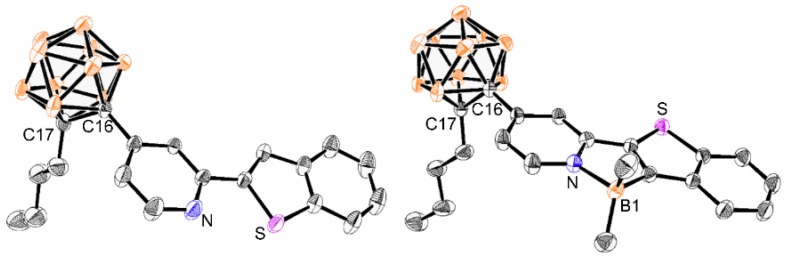
X-ray crystal structures of **CB2** (left) and **CB2B** (right) (50% thermal ellipsoids). H atoms are omitted for clarity.

All *o*-carboranyl compounds were fully confirmed by multinuclear (^1^H, ^13^C, and ^11^B) NMR spectroscopy (Figures S1–S7) and elemental analysis. The ^1^H and ^13^C NMR spectra of **CB1B** and **CB2B** show the expected resonances corresponding to (*C*^∧^*N*)BMe_2_. In particular, a characteristic singlet signal assignable to B−C*H*_3_ was detected at 0.05 and 0.15 ppm for **CB1B** and **CB2B**, respectively. Additionally, in the ^11^B NMR spectra of **CB1B** and **CB2B**, the shoulder signals in the region around δ 0 ppm and three broad peaks from δ −3 to −10 ppm clearly reveal the presence of tetracoordinated boron atoms and *closo*-*o*-carborane cage, respectively. The X-ray diffraction study revealed the molecular structures of **CB2** and **CB2B** in the solid state (Figure 2, detailed parameters and selected bond lengths and angles are listed in Tables S1 and S2). These results distinctly demonstrate that the *o*-carborane cage is appended at the C4 position of the pyridine ring of the btp moiety. In particular, the **CB2B** structure clearly exhibit a tetracoordinated structure with bidentate chelation by the boron atom.

### 3.2. Photophysical Properties

**Figure 3:**
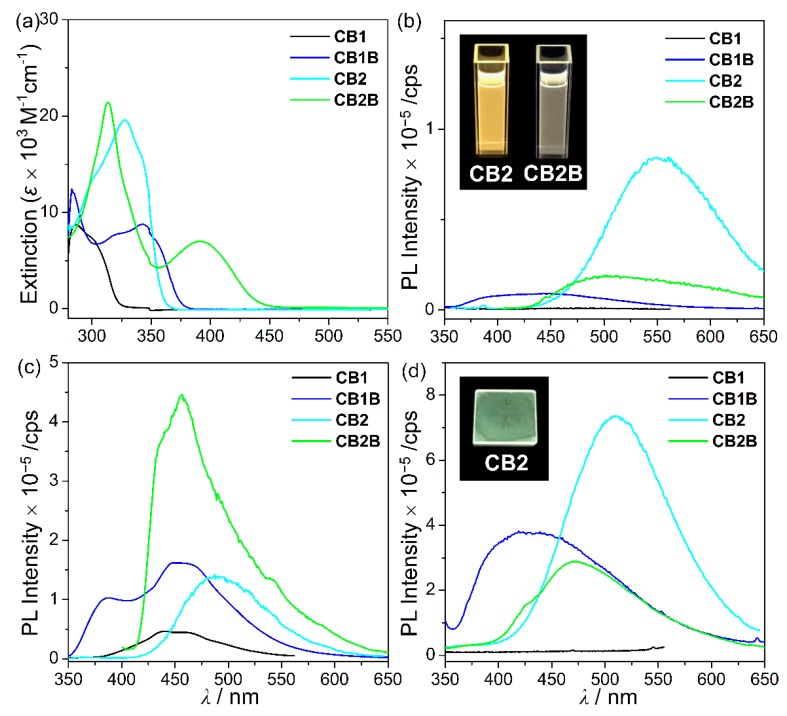
(a) UV-vis absorption spectra in toluene (5.0 × 10^−5^ M). Photoluminescence (PL) spectra in toluene (5.0 × 10^−5^ M) at (**b**) 298 and (**c**) 77 K. (**d**) PL spectra in film state (poly(methylmethacrylate) (PMMA) film doped with 5 wt % *o*-carbonyl compound). Inset figures show the color of the emission of each state under UV irradiation (*λ*_ex_ = 354 nm).

The photophysical properties of *o*-carboranyl compounds, **CB1**, **CB1B**, **CB2**, and **CB2B**, were investigated by UV-vis absorption and PL experiments (Figure 3). The non-chelated **CB1** and **CB2** display major absorption bands at *λ*_abs_ = 286 and 328 nm, respectively (Table 1). The broadness of these absorption bands strongly indicate that they can be assigned to not only a spin–allowed π-π * transition in the aryl groups (ppy or btp), but also ICT transition between the *o*-carborane and aryl groups. The absorption bands of the chelated **CB1B** and **CB2B**, which are assignable to a π-π * transition in the aryl groups, are also similarly exhibited in the high-energy region at *λ*_abs_ = 283 and 314 nm, respectively. Interestingly, **CB1B** and **CB2B** also show a dominant low-energy absorption band at *λ*_abs_ = 343 and 391 nm, respectively, which correlate with ICT from *o*-carborane to either the ppy or btp moiety (see calculated data below).

The fluorescent properties of the *o*-carboranyl compounds were further examined through PL measurements at various conditions (Figure 3b−d and Table 1). The emission spectra of **CB2** and **CB2B** in toluene (at 298 K) exhibit an intense emission at *λ*_em_ = 552 and 505 nm, respectively, that tail off to 650 nm. The emission spectrum of **CB1** does not show any peak, while that of **CB1B** show a faint emissive trace from 380 to 550 nm. On the other hand, the emission spectra of all compounds at 77 K are enhanced relative to those at 298 K. In particular, the emission spectra of **CB1B** shows a dual emissive pattern that can be divided to high- (above 450 nm) and low-energy (below 450 nm) regions, that of **CB2B** shows an intense emission from 430 to 600 nm, while those of **CB1** and **CB2** exhibit a single, broad emission band in the low-energy region. According to theoretical data, which will be discussed below, the low-energy emission of all compounds is closely associated with the ICT transition between the *o*-carborane and aryl groups, while the high-energy emission at 384 and 456 nm for **CB1B** and **CB2B**, respectively, closely involves π-π * transition in the BMe_2_-chelated aryl group. Thus, these features strongly indicate that radiative decay due to π-π * transition in the aryl groups and ICT transition associated with the *o*-carborane unit can be amplified in the rigid molecular state. These results are attributed to the inhibition of structural fluctuation, such as variation in the C−C bond in *o*-carborane and free rotation of the *o*-carborane cage [10,32,58]. Indeed, the calculated optimized structures of all compounds at the *S*_0_ and *S*_1_ states distinctly present evidence supporting structural fluctuation. Specifically, the C−C bond length (2.38–2.42 Å) in *o*-carborane at the *S*_1_ state (the structure that the one side of icosahedron is elongated) becomes significantly longer than that at the *S*_0_ state (≈ 1.73 Å) (Table 2), consistent with previous studies [10,39,58].

The most interesting feature is that **CB2** shows significant emission in solution at both 298 and 77 K, while **CB1** shows no emission in solution at 298 K and a weak emissive trace at 77 K. From these emissive characteristics, the *Φ*_em_ values of **CB1** and **CB2** in solution at 298 K are estimated to be <1% and 13%, respectively. The difference between the emissive properties of these non-chelated *o*-carboranyl compounds seem to be strongly correlated with their structure. Our group has already reported that radiative decay resulting from ICT transition in *o*-carboranes can be efficiently generated by maintaining the planarity of the aryl rings [58]. The optimized *S*_0_ and *S*_1_ structures of **CB2** specifically exhibit considerably similar dihedral angle between the pyridine and benzothiophene rings (*Ψ*_calc_ = 0.8° for *S*_0_ and 1.2° for *S*_1_), whereas those of **CB1** show significantly different *Ψ*_calc_ (22.2° for *S*_0_ and 0.3° for *S*_1_) (Table 2). The experimental dihedral angle for **CB2** (*Ψ*_exp_ = 2.9°), determined from the molecular structure at the solid state, is also similar to *Ψ*_calc_. These structural features clearly indicate that the planarity of the aryl groups of **CB2** can be sufficiently maintained in spite of repeated conversion between the *S*_0_ and excited states by an external energy. Because structural stability efficiently evokes radiative decay due to ICT transition involving *o*-carborane, **CB2** shows a high *Φ*_em_, moderate radiative decay constant (*k*_r_ = 2.2 × 10^7^ s^−1^), and low non-radiative decay constant (*k*_nr_ = 1.5 × 10^8^ s^−1^) in solution at 298 K (Table 1) [58]. The measured *Φ*_em_ of **CB1B** and **CB2B**, resulting from radiative decay due to π-π * transition in the aryl groups, are 3% and 6%, respectively, at 298 K.

The PL spectra for the film state (PMMA film doped with 5 wt% *o*-carboranyl compound) at 298 K exhibit emissive patterns similar to those in solution at 77 K (Figure 3d). The high-energy emissions of **CB1B** and **CB2B** corresponding to π-π * transition in the chelated aryl groups are still distinctively observed in the region centered at 424 and 473 nm, respectively, and extend to the low-energy emission region (ca. 600 nm) assigned to the ICT transition involving *o*-carborane. The estimated *Φ*_em_ of **CB1B** and **CB2B** are 8% and 9%, respectively, which are significantly enhanced compared with those in solution. These results arise from the efficient radiative mechanism induced by inhibition of structural fluctuation, such as elongation of the C−C bond and free rotation of the *o*-carborane cage, in the rigid solid state. Indeed, the *k*_nr_ values of both **CB1B** and **CB2B** in the film state increase by more than twice (1.6 × 10^7^ and 1.1 × 10^8^ s^−1^, respectively) as those in solution at 298 K.

Interestingly, the PL spectrum of **CB2** in the film state (Figure 3d inset) shows a *Φ*_em_ nearly twice enhanced (25%) as that in solution at 298 K; however, the *Φ*_em_ values of **CB1** in the solution and film states are similar. The emission band mainly involves ICT transition between *o*-carborane and aryl groups (see the calculated data below). The rigidity of both compounds can inhibit structural fluctuation, although the considerable difference between their emissive properties strongly reveal that maintaining the planarity of the aryl rings promotes radiative decay [58]. Consequently, the *k*_r_ of **CB2** in the film state (1.04 × 10^9^ s^−1^) is 50 times higher than that in solution (2.20 × 10^7^ s^−1^) at 298 K (Table 1).

### 3.3. Theoretical Calculations and Orbital Analysis

**Figure 4:**
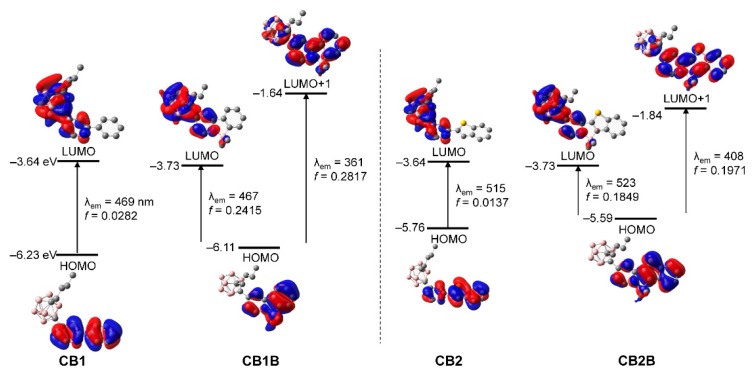
Relative energies of frontier molecular orbitals of *o*-carboranyl compounds at the first excited singlet state (*S*_1_) calculated by density functional theory (DFT) (isovalue = 0.04). The transition energy (in nm) was calculated at the TD-B3LYP/6-31G(d) level.

To elucidate the nature of the electronic transitions in the *o*-carboranyl compounds, TD-DFT optimization of the *S*_0_ and *S*_1_ structures of **CB1**, **CB2**, **CB1B**, and **CB2B** were carried out using the B3LYP functional (Figure 4 and Figure S11, Table 3 and Table S3). The calculated geometries were optimized from the X-ray crystal structures of **CB2** and **CB2B**. To include the effects of the toluene solvent [60,61], a conductor-like polarizable continuum model was also used. The computational data for the *S*_0_ state show that the lowest-energy electronic transition for the non-chelated compounds (**CB1** and **CB2**) is the highest occupied molecular orbital (HOMO) → lowest unoccupied molecular orbital (LUMO) transition (Figure S11 and Table S3). The HOMOs of both compounds are entirely localized on the aryl moiety (>99%, Tables S5 and S9), whereas the orbital contribution of the *o*-carborane unit to the LUMOs is slightly higher at >17%. These results indicate that the lowest energy absorption of **CB1** and **CB2** can be mainly attributed to π-π * transition in the aryl moieties, with a minor contribution from ICT transition between the *o*-carborane and aryl groups. On the other hand, the calculated data for the optimized *S*_0_ geometries of the chelated compounds (**CB1B** and **CB2B**) show that the lowest energy absorption mainly involves the two major transitions (*f*_calc_ > 0.12, Figure S11 and Table S3) from the HOMO to LUMO and LUMO + 1. The HOMO and LUMO + 1 levels of both compounds are predominantly localized over the BMe_2_-chelated aryl rings (>96%, Tables S7 and S11 in Supplementary Materials), whereas the LUMO is distributed over not only the aryl rings (∼84%), but also the *o*-carborane moiety (∼16%). These results suggest that the absorption spectra of **CB1B** and **CB2B** can be largely attributed to π-π * transition on the chelated aryl ring, with substantial contribution from the ICT transition associated with the *o*-carborane moiety, as in the non-chelated compounds. In addition, all calculated data for the optimized *S*_0_ structures match the experimental UV-vis absorption spectra well.

Based on the computational data for the *S*_1_ states of **CB1** and **CB2**, the major transition for the lowest-energy emission is the HOMO → LUMO transition (Figure 4 and Table 3). While the LUMOs of both compounds are significantly localized on the entire *o*-carborane moiety (∼85%, Tables S5 and S9), the HOMOs dominantly occupy the aryl groups (>99%). These results strongly suggest that the experimentally observed emission in the rigid states, namely the solution at 77 K and film (solid) state, dominantly originates from ICT between the *o*-carborane and aryl moieties. On the other hand, the major low-energy emissions of **CB1B** and **CB2B** are bipartitely assigned to the HOMO → LUMO and HOMO → LUMO+1 transitions (Figure 3 and Table 3). Although both the HOMO and LUMO+1 are mostly focused on the chelated aryl moieties (>90%, Tables S7 and S11), the LUMO has a significant contribution of around 77% from the *o*-carborane moiety. These results strongly suggest that the intense emissions in the high-energy region centered at ca. 380 nm for **CB1B** and ca. 450 nm for **CB2B** originate from π-π * transitions in the chelated aryl groups. Additionally, the tailed emission traces in the low-energy region below 500 nm are clearly attributed to the ICT transition from the *o*-carborane unit to the chelated aryl group. Consequently, all electronic transitions occurring in each *o*-carboranyl compound were precisely analyzed through theoretical calculation.

## 4. Conclusions

The ppy- and btp-based *o*-carboranyl compounds (**CB1** and **CB2**) and their BMe_2_-*C*^∧^*N* chelated compounds (**CB1B** and **CB2B**) were synthesized and fully characterized. The solid-state structures of **CB2** and **CB2B**, analyzed by single-crystal X-ray diffraction, clearly exhibited the *o*-carborane cage substituent at the C4 position for the pyridine ring and tetracoordinated dimethylboryl center of **CB2B**. **CB1B** and **CB2B** in the solution and film states demonstrated strong emission centered at ca. 450 and 500 nm, respectively, originating from π-π * transition in the aryl group; furthermore, the tailing off to 650 nm is attributed to ICT transition between the *o*-carborane and aryl groups. While **CB1** exhibited faint emissions in toluene solution at 298 K and the film state, **CB2** showed intense emissions in both states, which are assignable to radiative decay due to the ICT transition. On the other hand, the dihedral angle between the aromatic rings of **CB1** and **CB2** in the optimized *S*_0_ and *S*_1_ structures clearly revealed that the planarity of the btp groups of **CB2** could be maintained, while the ppy groups of **CB1** freely rotated from the ground to the excited states. These results distinctly suggest that the planarity of aryl groups appended to *o*-carborane have a decisive effect on the efficiency of the radiative decay due to the ICT transition.

## Figures and Tables

**Table 1:** Photophysical data of *o*-carboranyl compounds.

**Compound**	***λ*** **_abs_** **^1^/nm** **(ε × 10^−3^ M^−1^ cm^−1^)**		***λ*** **_ex_** **/nm**	***λ*** **_em_** **/nm**			***Φ*** **_em_** **^3^**	
				**298 K ^1^**	**77 K ^1^**	**Film ^2^**	**298 K ^1^**	**Film ^2^**
**CB1**	286 (8.8)		286	− ^4^	445	− ^4^	<0.01	<0.01
**CB1B**	283 (12.4), 343 (8.8)		337	451	384, 454	424	0.03	0.08
**CB2**	328 (19.6)		328	552	487	510	0.13	0.25
**CB2B**	314 (21.4), 391 (7.0)		391	505	456	473	0.06	0.09
**Compound**	**τ/ns**		***k*** **_r_** **^5^/** **× 10^8^** **s^−1^**			***k*** **_nr_** **^6^/** **× 10^8^** **s^−1^**		
	**298 K ^1^**	**Film ^2^**	**298 K ^1^**		**Film ^2^**	**298 K ^1^**		**Film ^2^**
**CB1**	− ^4^	− ^4^	-		-	-		-
**CB1B**	5.5	5.0	0.05		0.16	1.76		1.84
**CB2**	5.9	0.24	0.22		10.4	1.47		31.3
**CB2B**	1.2	0.83	0.50		1.1	7.8		11.0

^1^*c* = 5.0 × 10^−5^ M in toluene. ^2^ Measured in film state (5 wt % doped on PMMA) at 298 K. ^3^ Absolute PL quantum yield. ^4^ Not observed due to weak emission. ^5^ *k*_r_ = *Φ*_em_ /*τ*. ^6^ *k*_nr_ = *k*_r_(1/*Φ*_em_−1).

**Table 2:**
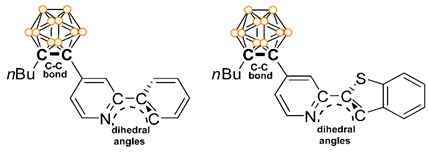
C−C bond length (Å) and N−C−C−C dihedral angle (*Ψ*, °) between aromatic rings (curved dotted lines in figure) in the optimized ground (*S*_0_) and first excited singlet state (*S*_1_) structures of *o*-carboranyl compounds

	CB1		CB1B		CB2		CB2B	
	*S*_0_	*S*_1_	*S*_0_	*S*_1_	*S*_0_	*S*_1_	*S*_0_	*S*_1_
C−C	1.72	2.39	1.73	2.42	1.72	2.38	1.74	2.40
*Ψ*_calc_	22.2	0.3	-	-	0.8	1.2	-	-
*Ψ*_exp_^1^	-		-		2.9		-	

^1^ Dihedral angle determined from the X-ray structure (Figure 2).

**Table 3:** Major low-energy electronic transitions in *o*-carborane compounds at the first excited singlet state (*S*_1_) calculated at the TD-B3LYP/6-31G(d) level ^1^.

	*λ*_calc_/nm	*f*_calc_	Assignment
CB1	468.98	0.0282	HOMO → LUMO (99.6%)
CB1B	466.86 360.99	0.2415 0.2817	HOMO → LUMO (99.6%) HOMO → LUMO+1 (74.8%)
CB2	515.13	0.0137	HOMO → LUMO (99.7%)
CB2B	522.90 433.83	0.1849 0.1971	HOMO → LUMO (99.8%) HOMO → LUMO+1 (70.2%)

^1^ Singlet energies for the vertical transition calculated using the optimized *S*_1_ geometries.

## Supplementary Materials

The following are available online at http://www.mdpi.com/1420-3049/24/1/201/s1, multinuclear NMR spectra (^1^H, ^13^C, and ^11^B) of *o*-carboranyl compounds (Figures S1–S7), X-ray crystallographic data in CIF format (Tables S1 and S2), and computational data (Figures S11–S15 and Tables S3–S19).
