# Supplementary material for: Effect of Planarity of Aromatic Rings Appended to o-Carborane on Photophysical Properties: A Series of o-Carboranyl Compounds Based on 2-Phenylpyridine- and 2-(Benzo[b]thiophen-2-yl)pyridine
Source: Molecules. 2019 Jan 7;24(1):201. doi: 10.3390/molecules24010201 (PMC6337515; doi:10.3390/molecules24010201)

checkCIF/PLATON report

Structure factors have been supplied for datablock(s) CB2

THIS REPORT IS FOR GUIDANCE ONLY. IF USED AS PART OF A REVIEW PROCEDURE FOR PUBLICATION, IT SHOULD NOT REPLACE THE EXPERTISE OF AN EXPERIENCED CRYSTALLOGRAPHIC REFEREE.

No syntax errors found. CIF dictionary Interpreting this report

Datablock: CB2

```
Bond precision:  C-C = 0.0041 A          Wavelength=0.71073

Cell:            a=14.7656(6)    b=11.0253(5)        c=14.9976(6)
                 alpha=90        beta=107.893(2)     gamma=90
Temperature:     296 K

                Calculated                Reported
Volume          2323.44(17)               2323.44(17)
Space group     P 21/c                    P 21/c
Hall group      : -P 2ybc                 -P 2ybc
Moiety formula  C19 H27 B10 N S           C19H27B10Ns
Sum formula     C19 H27 B10 N S           C19 H27 B10 N S
Mr              409.58                    409.57
Dx,g cm-3       1.171                     1.171
Z               4                         4
Mu (mm-1)       0.147                     0.147
F000            856.0                     856.0
F000'           856.68
h,k,lmax        17,13,18                  17,13,18
Nref            4365                      4340
Tmin,Tmax       0.971,0.978               0.971,0.978
Tmin'           0.971

Correction method= # Reported T Limits: Tmin=0.971 Tmax=0.978
AbsCorr = MULTI-SCAN

Data completeness= 0.994          Theta(max)= 25.569

R(reflections)= 0.0640( 3503)     wR2(reflections)= 0.1745( 4340)

S = 1.074                  Npar= 281
```

The following ALERTS were generated. Each ALERT has the format
test-name_ALERT_alert-type_alert-level.
Click on the hyperlinks for more details of the test.

Alert level B

```
PLAT919_ALERT_3_B Reflection # Likely Affected by the Beamstop ...          1 Check
```

Alert level C

```
PLAT220_ALERT_2_C Non-Solvent  Resd 1  C   Ueq(max)/Ueq(min) Range        3.2 Ratio
PLAT241_ALERT_2_C High   'MainMol' Ueq as Compared to Neighbors of        C13 Check
PLAT340_ALERT_3_C Low Bond Precision on  C-C Bonds ...............    0.00411 Ang.
PLAT906_ALERT_3_C Large K Value in the Analysis of Variance ......      9.823 Check
PLAT906_ALERT_3_C Large K Value in the Analysis of Variance ......      2.204 Check
PLAT918_ALERT_3_C Reflection(s) with I(obs) much Smaller I(calc) .          1 Check
PLAT934_ALERT_3_C Number of (Iobs-Icalc)/SigmaW > 10 Outliers ....          1 Check
```

Alert level G

```
PLAT042_ALERT_1_G Calc. and Reported MoietyFormula Strings  Differ     Please Check
PLAT066_ALERT_1_G Predicted and Reported Tmin&Tmax Range Identical          ? Check
PLAT343_ALERT_2_G Unusual sp?      Angle Range in Main Residue for        C16 Check
PLAT343_ALERT_2_G Unusual sp?      Angle Range in Main Residue for        C17 Check
PLAT367_ALERT_2_G Long?  C(sp?)-C(sp?) Bond  C11      - C16      .       1.51 Ang.
PLAT367_ALERT_2_G Long?  C(sp?)-C(sp?) Bond  C16      - C17      .       1.70 Ang.
PLAT367_ALERT_2_G Long?  C(sp?)-C(sp?) Bond  C17      - C18      .       1.53 Ang.
PLAT912_ALERT_4_G Missing # of FCF Reflections Above STh/L=  0.600         25 Note
PLAT978_ALERT_2_G Number C-C Bonds with Positive Residual Density.          4 Info
```

0 **ALERT level A** = Most likely a serious problem - resolve or explain
1 **ALERT level B** = A potentially serious problem, consider carefully
7 **ALERT level C** = Check. Ensure it is not caused by an omission or oversight
9 **ALERT level G** = General information/check it is not something unexpected

2 ALERT type 1 CIF construction/syntax error, inconsistent or missing data
8 ALERT type 2 Indicator that the structure model may be wrong or deficient
6 ALERT type 3 Indicator that the structure quality may be low
1 ALERT type 4 Improvement, methodology, query or suggestion
0 ALERT type 5 Informative message, check

It is advisable to attempt to resolve as many as possible of the alerts in all categories. Often the minor alerts point to easily fixed oversights, errors and omissions in your CIF or refinement strategy, so attention to these fine details can be worthwhile. In order to resolve some of the more serious problems it may be necessary to carry out additional measurements or structure refinements. However, the purpose of your study may justify the reported deviations and the more serious of these should normally be commented upon in the discussion or experimental section of a paper or in the "special_details" fields of the CIF. checkCIF was carefully designed to identify outliers and unusual parameters, but every test has its limitations and alerts that are not important in a particular case may appear. Conversely, the absence of alerts does not guarantee there are no aspects of the results needing attention. It is up to the individual to critically assess their own results and, if necessary, seek expert advice.

Publication of your CIF in IUCr journals

A basic structural check has been run on your CIF. These basic checks will be run on all CIFs submitted for publication in IUCr journals (*Acta Crystallographica*, *Journal of Applied Crystallography*, *Journal of Synchrotron Radiation*); however, if you intend to submit to *Acta Crystallographica Section C* or *E* or *IUCrData*, you should make sure that full publication checks are run on the final version of your CIF prior to submission.

Publication of your CIF in other journals

Please refer to the *Notes for Authors* of the relevant journal for any special instructions relating to CIF submission.

PLATON version of 19/10/2018; check.def file version of 15/10/2018

Datablock CB2 - ellipsoid plot

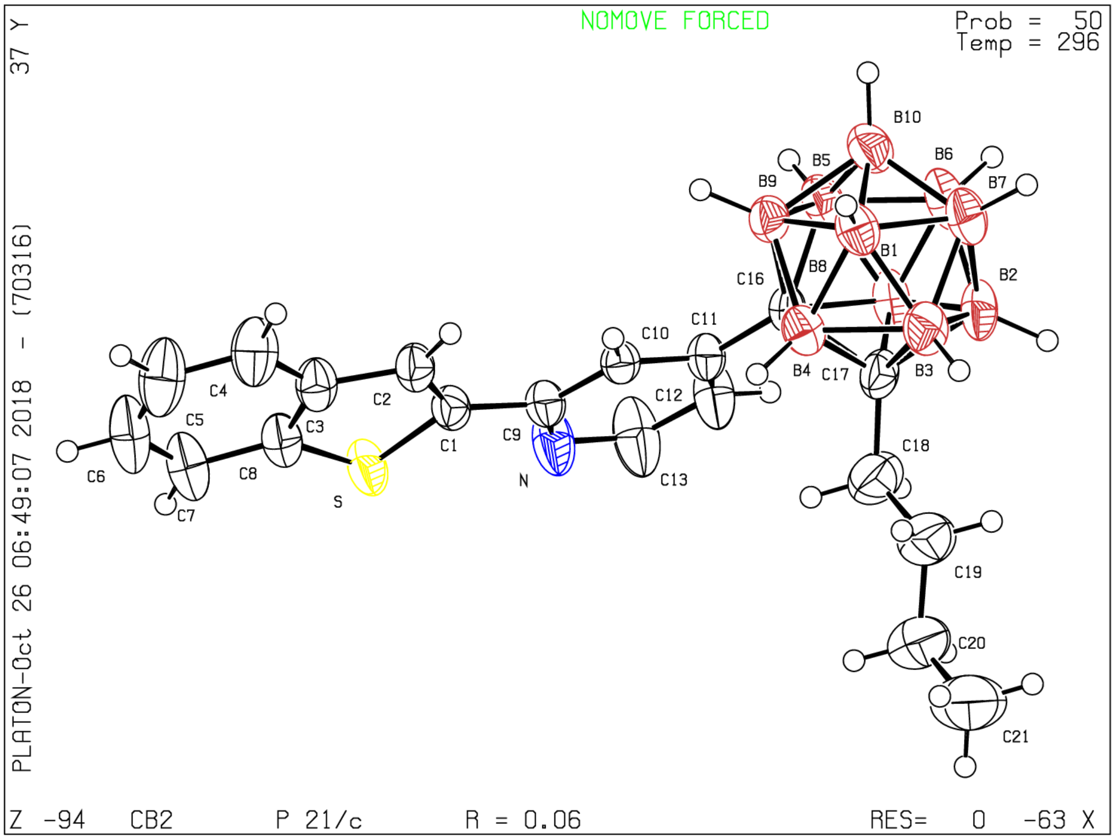

Supplement: Supplementary file 1 [file molecules-24-00201-s001.zip › Supplementary files/checkcif[CB2].pdf]
