# Supplementary material for: Effect of Planarity of Aromatic Rings Appended to o-Carborane on Photophysical Properties: A Series of o-Carboranyl Compounds Based on 2-Phenylpyridine- and 2-(Benzo[b]thiophen-2-yl)pyridine
Source: Molecules. 2019 Jan 7;24(1):201. doi: 10.3390/molecules24010201 (PMC6337515; doi:10.3390/molecules24010201)

checkCIF/PLATON report

You have not supplied any structure factors. As a result the full set of tests cannot be run.

No syntax errors found.   CIF dictionary   Interpreting this report

Datablock: kaist04

Bond precision: C-C = 0.0038 A   Wavelength=0.71073

Cell:	a=13.576(5)	b=14.325(5)	c=13.570(4)
	alpha=90	beta=101.03(2)	gamma=90
Temperature:	296 K		

	Calculated	Reported
Volume	2590.3(15)	2590.3(15)
Space group	P 21/c	P2(1)/c
Hall group	-P 2ybc	?
Moiety formula	C21 H32 B11 N S	C21H32B11N1S1
Sum formula	C21 H32 B11 N S	C21 H32 B11 N S
Mr	449.46	449.45
Dx,g cm-3	1.153	1.152
Z	4	4
Mu (mm-1)	0.137	0.137
F000	944.0	944.0
F000’	944.70	
h,k,lmax	16,17,16	16,17,16
Nref	4569	4548
Tmin,Tmax	0.984,0.993	0.930,0.941
Tmin’	0.960	

Correction method= MULTI-SCAN

Data completeness= 0.995   Theta(max)= 25.000

R(reflections)= 0.0500(2869)   wR2(reflections)= 0.1596(4548)

S = 1.029   Npar= 310

The following ALERTS were generated. Each ALERT has the format
test-name_ALERT_alert-type_alert-level.
Click on the hyperlinks for more details of the test.

Alert level C

PLAT367_ALERT_2_C Long? C(sp?)-C(sp?) Bond C16 - C17 ... 1.69 Ang.

Alert level G

```
PLAT005_ALERT_5_G No _iucr_refine_instructions_details  in the CIF          ?
PLAT042_ALERT_1_G Calc. and Reported MoietyFormula Strings  Differ         ?
PLAT093_ALERT_1_G No su’s on H-positions, refinement reported as .      mixed
PLAT343_ALERT_2_G Check sp?     Angle Range in Main Residue for ..        C16
PLAT343_ALERT_2_G Check sp?     Angle Range in Main Residue for ..        C17
```

```
   0 ALERT level A = Most likely a serious problem - resolve or explain
   0 ALERT level B = A potentially serious problem, consider carefully
   1 ALERT level C = Check. Ensure it is not caused by an omission or oversight
   5 ALERT level G = General information/check it is not something unexpected

   2 ALERT type 1 CIF construction/syntax error, inconsistent or missing data
   3 ALERT type 2 Indicator that the structure model may be wrong or deficient
   0 ALERT type 3 Indicator that the structure quality may be low
   0 ALERT type 4 Improvement, methodology, query or suggestion
   1 ALERT type 5 Informative message, check
```

It is advisable to attempt to resolve as many as possible of the alerts in all categories. Often the minor alerts point to easily fixed oversights, errors and omissions in your CIF or refinement strategy, so attention to these fine details can be worthwhile. In order to resolve some of the more serious problems it may be necessary to carry out additional measurements or structure refinements. However, the purpose of your study may justify the reported deviations and the more serious of these should normally be commented upon in the discussion or experimental section of a paper or in the "special_details" fields of the CIF. checkCIF was carefully designed to identify outliers and unusual parameters, but every test has its limitations and alerts that are not important in a particular case may appear. Conversely, the absence of alerts does not guarantee there are no aspects of the results needing attention. It is up to the individual to critically assess their own results and, if necessary, seek expert advice.

Publication of your CIF in IUCr journals

A basic structural check has been run on your CIF. These basic checks will be run on all CIFs submitted for publication in IUCr journals (*Acta Crystallographica*, *Journal of Applied Crystallography*, *Journal of Synchrotron Radiation*); however, if you intend to submit to *Acta Crystallographica Section C* or *E*, you should make sure that full publication checks are run on the final version of your CIF prior to submission.

Publication of your CIF in other journals

Please refer to the *Notes for Authors* of the relevant journal for any special instructions relating to CIF submission.

PLATON version of 05/11/2012; check.def file version of 05/11/2012

Datablock kaist04 - ellipsoid plot

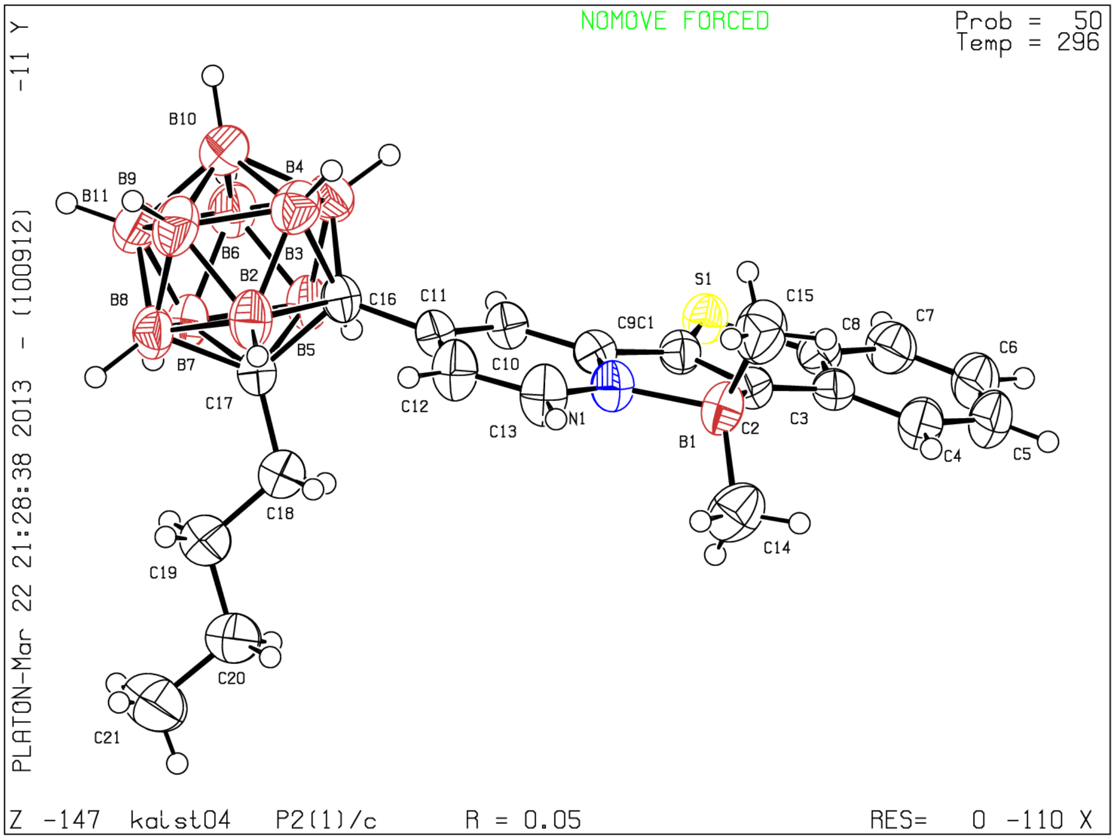

Supplement: Supplementary file 1 [file molecules-24-00201-s001.zip › Supplementary files/checkcif[CB2B].pdf]
