# Supplementary material for: Effect of Planarity of Aromatic Rings Appended to o-Carborane on Photophysical Properties: A Series of o-Carboranyl Compounds Based on 2-Phenylpyridine- and 2-(Benzo[b]thiophen-2-yl)pyridine
Source: Molecules. 2019 Jan 7;24(1):201. doi: 10.3390/molecules24010201 (PMC6337515; doi:10.3390/molecules24010201)
Supplement: Supplementary file 1 [file molecules-24-00201-s001.zip › Supplementary files/SM-4CBBMe2-Mole.doc]

**Supplementary Materials**

**Effect of Planarity of Aromatic Rings Appended to *o*-Carborane on Photophysical Properties: A Series of *o*-Carboranyl Compounds Based on 2-Phenylpyridine- and 2-(Benzo[*b*]thiophen-2-yl)pyridine**

**Hyomin Jin ^1,†^_,_ Seonah Kim ^1,†^, Hye Jin Bae ^2^, Ji Hye Lee ^1^, Hyonseok Hwang ^1^, Myung Hwan Park ^3,^* and Kang Mun Lee ^1,^***

^1^ Department of Chemistry, Institute for Molecular Science and Fusion Technology,

Kangwon National University, Chuncheon 24341, Republic of Korea

^2^ Department of Chemistry, KAIST, Daejeon 34142, Republic of Korea.

^3^ Department of Chemistry Education, Chungbuk National University,

Cheongju 28644, Republic of Korea

*

**Figure S1.** ^1^H (top) and ^13^C (bottom) NMR spectra of **3** (* from residual CHCl_3_ in CDCl_3_)

*

**Figure S2.** ^1^H (top) and ^13^C (bottom) NMR spectra of **1a** (* from residual CHCl_3_ in CDCl_3_).

*

**Figure S3.** ^1^H (top) and ^13^C (bottom) NMR spectra of **2a** (* from residual CHCl_3_ in CDCl_3_).

*

**Figure S4.** ^1^H (top), ^13^C (middle) and ^11^B (bottom) NMR spectra of **CB1** (* from residual CHCl_3_ in CDCl_3_).

*

**Figure S5.** ^1^H (top), ^13^C (middle) and ^11^B (bottom) NMR spectra of **CB2** (* from residual CHCl_3_ in CDCl_3_).

*

**Figure S6.** ^1^H (top), ^13^C (middle) and ^11^B (bottom) NMR spectra of **CB1B** (* from residual CHCl_3_ in CDCl_3_).

*

**Figure S7.** ^1^H (top), ^13^C (middle) and ^11^B (bottom) NMR spectra of **CB2B** (* from residual CHCl_3_ in CDCl_3_).

**Table S1.** Crystallographic data and parameters for **CB2** and **CB2B**

| Compound | **CB2** | **CB2B** |
| --- | --- | --- |
| Formula | C_19_H_27_B_10_NS | C_21_H_32_B_11_NS |
| Formula weight | 409.57 | 449.45 |
| Crystal system | Monoclinic | Monoclinic |
| Space group | P2_1_/c | P2_1_/c |
| *a* (Å) | 14.7656(6) | 13.576(5) |
| *b* (Å) | 11.0253(5) | 14.325(5) |
| *c* (Å) | 14.9976(6) | 13.570(4) |
| *α* (°) | 90 | 90 |
| *β* (°) | 107.893(2) | 101.03(2) |
| *γ* (°) | 90 | 90 |
| *V* (Å^3^) | 2323.44(17) | 2590.3(15) |
| *Z* | 4 | 4 |
| *ρ*_calc_(g cm^-3^) | 1.171 | 1.152 |
| *μ* (mm^-1^) | 0.147 | 0.137 |
| *F*(000) | 856 | 944 |
| *T* (K) | 296(2) | 296(2) |
| Scan mode | *multi-scan* | *multi-scan* |
| *hkl* range | −17 < *h* < 17,  −13 < *k* < 13,  −18 < *l* < 18 | −16 < *h* < 14,  −15 < *k* < 17,  −15 < *l* < 16 |
| Measd reflns | 61389 | 20372 |
| Unique reflns [*R*_int_] | 4340 [R(int) = 0.0700] | 4548 [R(int) = 0.0477] |
| Reflns used for refinement | 4340 | 4548 |
| Refined parameters | 281 | 310 |
| *R*_1_*^a^* (I > 2σ(I)) | 0.0640 | 0.0500 |
| *wR*_2_*^b^* all data | 0.1745 | 0.1596 |
| GOF on *F*^2^ | 1.074 | 1.029 |
| *ρ*_fin_ (max/min) (e Å^−3^) | 0.371, −0.224 | 0.208, −0.217 |

*^a^*R_1_ = ∑||*F*o| − |*F*c||/∑|*F*o|. *^b^w*R_2_ = {[∑*w*(*F*o^2^ − *F*c^2^)^2^]/[∑*w*(*F*o^2^)^2^]}^1/2^.

**Table S2.** Selected bond lengths (Å) for **CB2** and **CB2B**

| Compound | **CB2** | **CB2B** |
| --- | --- | --- |
| B1‒N | ‒ | 1.653(3) |
| B1‒C2 | ‒ | 1.595(4) |
| B1‒C14 | ‒ | 1.617(4) |
| B1‒C15 | ‒ | 1.608(4) |
| C16‒C17  (Carborane C‒C) | 1.699(3) | 1.694(3) |

**Figure S8.** Emission decay curves for **CB1B** in (a) toluene (5.0 × 10^–5^ M) and (b) film (5 wt% doped on PMMA) detected at each maximum wavelength point, and these exponential fitting curves (red-line, single exponential curve fittings, respectively).

**Figure S9.** Emission decay curves for **CB2** in (a) toluene (5.0 × 10^–5^ M) and (b) film (5 wt% doped on PMMA) detected at each maximum wavelength point, and these exponential fitting curves (red-line, single and double exponential curve fittings, respectively).

**Figure S10.** Emission decay curves for **CB2B** in (a) toluene (5.0 × 10^–5^ M) and (b) film (5 wt% doped on PMMA) detected at each maximum wavelength point, and these exponential fitting curves (red-line, single and double exponential curve fittings, respectively).

**Computational details**

**Figure S11.** Frontier molecular orbitals for *o*-carboranyl complexes at their ground state (S_0_) with their relative energies from DFT calculation (isovalue 0.04). The transition energy (in nm) was calculated using the TD-B3LYP method with 6-31G(d) basis sets.

**Table S3.** The major low-energy electronic transition for *o*-carborane compounds at their ground state (S_0_) calculated using the TD-B3LYP method with 6-31G(d) basis sets.

|  | λ_calc_/ nm | *f*_calc_ | Assignment |
| --- | --- | --- | --- |
| **CB1** | 293.41 | 0.2248 | HOMO → LUMO (93.0%) |
| **CB1B** | 350.58  293.13 | 0.1254  0.1433 | HOMO → LUMO (97.9%)  HOMO → LUMO+1 (88.4%) |
| **CB2** | 346.39 | 0.3498 | HOMO → LUMO (85.7%) |
| **CB2B** | 416.18  330.50 | 0.2523  0.4642 | HOMO → LUMO (97.4%)  HOMO → LUMO+1 (89.6%) |

| S_0_ |  |  |  |  |
| --- | --- | --- | --- | --- |
|  | LUMO+3 | LUMO+2 | LUMO+1 | LUMO |
|  |  |  |  |  |
|  | HOMO | HOMO‒1 | HOMO‒2 | HOMO‒3 |
| S_1_ |  |  |  |  |
|  | LUMO+3 | LUMO+2 | LUMO+1 | LUMO |
|  |  |  |  |  |
|  | HOMO | HOMO‒1 | HOMO‒2 | HOMO‒3 |

**Figure S12.** The selected frontier orbitals of **CB1** from B3LYP calculations (Isovalue = 0.04 a.u.) at the ground state (S_0_) and first singlet excited state (S_1_) optimized geometries in toluene.

**Table S4.** Computed absorption wavelengths (*λ*_calc_ in nm) and oscillator strengths (*f*_calc._) for **CB1** from TD-B3LYP calculations using the B3LYP geometries at the ground state (S_0_) and first singlet excited state (S_1_) optimized geometries in toluene

| state | *λ*_calc_ (/nm) | *f*_calc_ | Major contribution | | |
| --- | --- | --- | --- | --- | --- |
| S_0_ | | | | | |
| 1 | 293.41 | 0.2248 | HOMO | → | LUMO (93.0%) |
| 2 | 278.13 | 0.0056 | HOMO−1 | → | LUMO (21.5%) |
|  |  |  | HOMO−2 | → | LUMO (75.3%) |
| 3 | 268.65 | 0.0306 | HOMO−1 | → | LUMO (62.8%) |
|  |  |  | HOMO−2 | → | LUMO (21.7%) |
| 4 | 258.77 | 0.1630 | HOMO | → | LUMO+1 (82.5%) |
|  |  |  | HOMO−1 | → | LUMO (8.2%) |
| S_1_ | | | | | |
| 1 | 468.98 | 0.0282 | HOMO | → | LUMO (99.6%) |
| 2 | 411.26 | 0.0080 | HOMO−1 | → | LUMO (99.6%) |
| 3 | 395.24 | 0.0003 | HOMO−2 | → | LUMO (93.8%) |
| 4 | 386.78 | 0.0010 | HOMO−3 | → | LUMO (25.8%) |
|  |  |  | HOMO−4 | → | LUMO (66.1%) |

**Table S5.** Molecular orbital energies (in eV) and molecular orbital distributions (in %) of **CB1** at the ground state (S_0_) and first singlet excited state (S_1_) optimized geometries in toluene

|  | E (eV) | *n*Bu+Carborane | ppy |
| --- | --- | --- | --- |
| S_0_ | | | |
| LUMO+2 | −0.32 | 96.9 | 3.1 |
| LUMO+1 | −1.23 | 9.7 | 90.3 |
| LUMO | −1.74 | 17.5 | 82.5 |
| HOMO | −6.51 | 0.4 | 99.6 |
| HOMO−1 | −6.98 | 0.0 | 100.0 |
| HOMO−2 | −7.41 | 1.4 | 98.5 |
| S_1_ | | | |
| LUMO+2 | −0.85 | 10.9 | 89.1 |
| LUMO+1 | −1.60 | 4.1 | 95.9 |
| LUMO | −3.64 | 85.8 | 14.2 |
| HOMO | −6.23 | 0.4 | 99.6 |
| HOMO−1 | −7.08 | 0.1 | 99.9 |
| HOMO−2 | −7.45 | 1.4 | 98.6 |

| S_0_ |  |  |  |  |
| --- | --- | --- | --- | --- |
|  | LUMO+3 | LUMO+2 | LUMO+1 | LUMO |
|  |  |  |  |  |
|  | HOMO | HOMO‒1 | HOMO‒2 | HOMO‒3 |
| S_1_ |  |  |  |  |
|  | LUMO+3 | LUMO+2 | LUMO+1 | LUMO |
|  |  |  |  |  |
|  | HOMO | HOMO‒1 | HOMO‒2 | HOMO‒3 |

**Figure S13.** The selected frontier orbitals of **CB1B** from B3LYP calculations (Isovalue = 0.04 a.u.) at the ground state (S_0_) and first singlet excited state (S_1_) optimized geometries in toluene.

**Table S6.** Computed absorption wavelengths (*λ*_calc_ in nm) and oscillator strengths (*f*_calc._) for **CB1B** from TD-B3LYP calculations using the B3LYP geometries at the ground state (S_0_) and first singlet excited state (S_1_) optimized geometries in toluene

| state | *λ*_calc_ (/nm) | *f*_calc_ | Major contribution | | |
| --- | --- | --- | --- | --- | --- |
| S_0_ | | | | | |
| 1 | 350.68 | 0.1254 | HOMO | → | LUMO (97.9%) |
| 2 | 322.83 | 0.0874 | HOMO−1 | → | LUMO (96.8%) |
| 3 | 293.13 | 0.1433 | HOMO | → | LUMO+1 (88.4%) |
| 4 | 282.96 | 0.0070 | HOMO−2 | → | LUMO (97.7%) |
| S_1_ | | | | | |
| 1 | 466.86 | 0.2415 | HOMO | → | LUMO (99.6%) |
| 2 | 394.97 | 0.0020 | HOMO−1 | → | LUMO (99.4%) |
| 3 | 381.34 | 0.0200 | HOMO−2 | → | LUMO (99.2%) |
| 4 | 360.99 | 0.2817 | HOMO | → | LUMO+1 (74.8%) |
|  |  |  | HOMO−3 | → | LUMO (9.9%) |

**Table S7.** Molecular orbital energies (in eV) and molecular orbital distributions (in %) of **CB1B** at the ground state (S_0_) and first singlet excited state (S_1_) optimized geometries in toluene

|  | E (eV) | *n*Bu+Carborane | ppy+BMe_2_ |
| --- | --- | --- | --- |
| S_0_ | | | |
| LUMO+2 | −0.41 | 97.8 | 2.2 |
| LUMO+1 | −1.51 | 3.1 | 96.8 |
| LUMO | −2.21 | 15.6 | 84.4 |
| HOMO | −6.34 | 0.3 | 99.7 |
| HOMO−1 | −6.62 | 0.2 | 99.8 |
| HOMO−2 | −7.40 | 0.0 | 100 |
| S_1_ | | | |
| LUMO+2 | −1.35 | 8.9 | 91.1 |
| LUMO+1 | −1.64 | 10.2 | 90.8 |
| LUMO | −3.73 | 77.8 | 22.2 |
| HOMO | −6.11 | 0.4 | 99.6 |
| HOMO−1 | −6.68 | 0.3 | 99.7 |
| HOMO−2 | −7.36 | 0.0 | 100 |

| S_0_ |  |  |  |  |
| --- | --- | --- | --- | --- |
|  | LUMO+3 | LUMO+2 | LUMO+1 | LUMO |
|  |  |  |  |  |
|  | HOMO | HOMO‒1 | HOMO‒2 | HOMO‒3 |
| S_1_ |  |  |  |  |
|  | LUMO+3 | LUMO+2 | LUMO+1 | LUMO |
|  |  |  |  |  |
|  | HOMO | HOMO‒1 | HOMO‒2 | HOMO‒3 |

**Figure S14.** The selected frontier orbitals of **CB2** from B3LYP calculations (Isovalue = 0.04 a.u.) at the ground state (S_0_) and first singlet excited state (S_1_) optimized geometries in toluene.

**Table S8.** Computed absorption wavelengths (*λ*_calc_ in nm) and oscillator strengths (*f*_calc._) for **CB2** from TD-B3LYP calculations using the B3LYP geometries at the ground state (S_0_) and first singlet excited state (S_1_) optimized geometries in toluene

| state | *λ*_calc_ (/nm) | *f*_calc_ | Major contribution | | |
| --- | --- | --- | --- | --- | --- |
| S_0_ | | | | | |
| 1 | 346.39 | 0.3498 | HOMO | → | LUMO (95.5%) |
| 2 | 319.07 | 0.0209 | HOMO−1 | → | LUMO (87.3%) |
| 3 | 301.76 | 0.0927 | HOMO | → | LUMO+1 (85.7%) |
| 4 | 281.28 | 0.0021 | HOMO−2 | → | LUMO (97.5%) |
| S_1_ | | | | | |
| 1 | 515.13 | 0.0137 | HOMO | → | LUMO (99.7%) |
| 2 | 496.92 | 0.0004 | HOMO−1 | → | LUMO (99.3%) |
| 3 | 383.00 | 0.0012 | HOMO−2 | → | LUMO (90.5%) |
| 4 | 374.26 | 0.0008 | HOMO−3 | → | LUMO (90.5%) |

**Table S9.** Molecular orbital energies (in eV) and molecular orbital distributions (in %) of **CB2** at the ground state (S_0_) and first singlet excited state (S_1_) optimized geometries in toluene

|  | E (eV) | *n*Bu+Carborane | btp |
| --- | --- | --- | --- |
| S_0_ | | | |
| LUMO+2 | −0.33 | 96.8 | 3.2 |
| LUMO+1 | −1.42 | 16.8 | 83.2 |
| LUMO | −1.99 | 17.1 | 83.9 |
| HOMO | −6.00 | 0.2 | 99.8 |
| HOMO−1 | −6.43 | 0.1 | 99.9 |
| HOMO−2 | −7.55 | 1.6 | 98.4 |
| S_1_ | | | |
| LUMO+2 | −0.90 | 13.2 | 86.8 |
| LUMO+1 | −1.86 | 3.9 | 96.1 |
| LUMO | −3.64 | 83.5 | 16.5 |
| HOMO | −5.76 | 0.2 | 99.8 |
| HOMO−1 | −6.55 | 0.1 | 99.9 |
| HOMO−2 | −7.65 | 1.6 | 98.4 |

| S_0_ |  |  |  |  |
| --- | --- | --- | --- | --- |
|  | LUMO+3 | LUMO+2 | LUMO+1 | LUMO |
|  |  |  |  |  |
|  | HOMO | HOMO‒1 | HOMO‒2 | HOMO‒3 |
| S_1_ |  |  |  |  |
|  | LUMO+3 | LUMO+2 | LUMO+1 | LUMO |
|  |  |  |  |  |
|  | HOMO | HOMO‒1 | HOMO‒2 | HOMO‒3 |

**Figure S15.** The selected frontier orbitals of **CB2B** from B3LYP calculations (Isovalue = 0.04 a.u.) at the ground state (S_0_) and first singlet excited state (S_1_) optimized geometries in toluene.

**Table S10.** Computed absorption wavelengths (*λ*_calc_ in nm) and oscillator strengths (*f*_calc._) for **CB2B** from TD-B3LYP calculations using the B3LYP geometries at the ground state (S_0_) and first singlet excited state (S_1_) optimized geometries in toluene

| state | *λ*_calc_ (/nm) | *f*_calc_ | Major contribution | | |
| --- | --- | --- | --- | --- | --- |
| S_0_ | | | | | |
| 1 | 416.18 | 0.2523 | HOMO | → | LUMO (97.4%) |
| 2 | 346.89 | 0.0894 | HOMO−1 | → | LUMO (93.1%) |
| 3 | 330.50 | 0.4642 | HOMO | → | LUMO+1 (89.6%) |
| 4 | 292.27 | 0.1795 | HOMO−1 | → | LUMO+1 (92.2%) |
| S_1_ | | | | | |
| 1 | 522.90 | 0.1849 | HOMO | → | LUMO (99.8%) |
| 2 | 459.03 | 0.0081 | HOMO−1 | → | LUMO (99.6%) |
| 3 | 434.10 | 0.1270 | HOMO−2 | → | LUMO (98.7%) |
| 4 | 433.83 | 0.1971 | HOMO | → | LUMO+1 (70.2%) |
|  |  |  | HOMO−2 | → | LUMO (13.2%) |

**Table S11.** Molecular orbital energies (in eV) and molecular orbital distributions (in %) of **CB2B** at the ground state (S_0_) and first singlet excited state (S_1_) optimized geometries in toluene

|  | E (eV) | *n*Bu+Carborane | btp+BMe_2_ |
| --- | --- | --- | --- |
| S_0_ | | | |
| LUMO+2 | −0.40 | 95.7 | 4.2 |
| LUMO+1 | −1.59 | 3.3 | 96.7 |
| LUMO | −2.29 | 15.6 | 84.4 |
| HOMO | −5.80 | 0.2 | 99.8 |
| HOMO−1 | −6.40 | 0.1 | 99.9 |
| HOMO−2 | −7.27 | 1.0 | 99 |
| S_1_ | | | |
| LUMO+2 | −1.36 | 12.9 | 87.1 |
| LUMO+1 | −1.84 | 10.0 | 90.1 |
| LUMO | −3.73 | 77.1 | 22.9 |
| HOMO | −5.59 | 0.3 | 99.7 |
| HOMO−1 | −6.50 | 0.1 | 99.9 |
| HOMO−2 | −7.24 | 1.7 | 98.3 |

**Table S12.** Cartesian coordinates of the ground state (S_0_) fully optimized geometry in toluene of **CB1** from B3LYP calculations (in Å)

| Atom | x | y | z |
| --- | --- | --- | --- |
| C | 2.302926 | 0.252033 | -0.071734 |
| H | 4.666288 | -2.606737 | -1.074809 |
| H | 2.528913 | -0.752602 | -2.344287 |
| H | 4.679254 | 0.470490 | -0.737993 |
| H | 5.279376 | -1.407516 | 1.690570 |
| H | 1.663788 | -3.367488 | -1.116443 |
| H | 3.503005 | 1.122803 | 1.936269 |
| H | 3.379615 | -3.813900 | 1.450107 |
| H | 0.656211 | 0.307389 | 1.800333 |
| H | 0.471528 | -2.691225 | 1.529508 |
| H | 2.676938 | -1.486359 | 3.332589 |
| B | 2.655375 | -1.007334 | -1.198970 |
| B | 3.856517 | -2.035720 | -0.418664 |
| B | 3.920785 | -0.269629 | -0.205915 |
| B | 2.151078 | -2.529216 | -0.435818 |
| B | 4.204400 | -1.337682 | 1.187140 |
| B | 3.217162 | 0.123985 | 1.371474 |
| B | 3.106315 | -2.728580 | 1.048388 |
| B | 1.513536 | -0.354886 | 1.331229 |
| B | 1.437996 | -2.124090 | 1.144996 |
| B | 2.696485 | -1.382267 | 2.148918 |
| C | 1.819911 | 1.580086 | -0.667043 |
| H | 2.218950 | 1.658466 | -1.683524 |
| H | 0.729622 | 1.538729 | -0.762004 |
| C | 2.203868 | 2.836582 | 0.127538 |
| H | 3.294947 | 2.897618 | 0.223582 |
| H | 1.800559 | 2.771005 | 1.145827 |
| C | 1.679372 | 4.111021 | -0.548422 |
| H | 2.076217 | 4.169451 | -1.571338 |
| H | 0.586670 | 4.047711 | -0.645374 |
| C | 2.053261 | 5.382666 | 0.219617 |
| H | 3.141674 | 5.489315 | 0.302800 |
| H | 1.667215 | 6.276156 | -0.283550 |
| H | 1.641664 | 5.366711 | 1.236097 |
| C | -0.107721 | -0.943096 | -0.859241 |
| C | -0.281503 | -1.159753 | -2.231176 |
| C | -1.242144 | -0.638140 | -0.102137 |
| C | -1.563114 | -1.035076 | -2.765135 |
| H | 0.545832 | -1.421408 | -2.877823 |
| C | -2.500480 | -0.550193 | -0.721371 |
| H | -1.154494 | -0.456509 | 0.960027 |
| H | -1.719405 | -1.189664 | -3.830851 |
| C | -3.729139 | -0.229734 | 0.053505 |
| C | -3.814670 | -0.463593 | 1.436609 |
| C | -6.002684 | 0.634280 | 0.101086 |
| C | -4.976390 | -0.149098 | 2.141354 |
| H | -2.984329 | -0.918164 | 1.968345 |
| C | -6.073521 | 0.404540 | 1.477847 |
| H | -6.851511 | 1.063813 | -0.423985 |
| H | -5.025467 | -0.344606 | 3.208909 |
| H | -6.977442 | 0.650802 | 2.028011 |
| C | 1.247861 | -1.095518 | -0.206368 |
| N | -2.648286 | -0.737503 | -2.047376 |
| C | -4.844105 | 0.316925 | -0.604868 |
| H | -4.786229 | 0.491748 | -1.673633 |

**Table S13.** Cartesian coordinates of the first excited state (S_1_) fully optimized geometry in toluene of **CB1** from B3LYP calculations (in Å)

| Atom | x | y | z |
| --- | --- | --- | --- |
| C | 2.675568 | 0.276705 | -0.187392 |
| H | 4.287340 | -3.136770 | -0.827915 |
| H | 2.272906 | -1.145398 | -2.198062 |
| H | 4.916420 | -0.253029 | -0.916703 |
| H | 5.174667 | -1.740053 | 1.789341 |
| H | 1.390966 | -3.685765 | -0.679506 |
| H | 3.834137 | 1.033691 | 1.809250 |
| H | 3.047566 | -3.810299 | 1.930547 |
| H | 0.807841 | 0.603468 | 1.445580 |
| H | 0.305354 | -2.393254 | 2.024056 |
| H | 2.594599 | -1.111916 | 3.379298 |
| B | 2.398445 | -1.201553 | -1.013544 |
| B | 3.584364 | -2.374274 | -0.237172 |
| B | 4.006990 | -0.653498 | -0.258202 |
| B | 1.866595 | -2.746531 | -0.120768 |
| B | 4.114946 | -1.553782 | 1.271320 |
| B | 3.397214 | 0.074551 | 1.257358 |
| B | 2.855168 | -2.781475 | 1.355965 |
| B | 1.571411 | -0.213917 | 1.032221 |
| B | 1.252233 | -2.015695 | 1.406468 |
| B | 2.605543 | -1.206158 | 2.189381 |
| C | 2.457479 | 1.568153 | -0.954600 |
| H | 3.086454 | 1.549669 | -1.854799 |
| H | 1.419119 | 1.588537 | -1.318088 |
| C | 2.728611 | 2.864051 | -0.176040 |
| H | 3.778337 | 2.885947 | 0.144833 |
| H | 2.125787 | 2.874245 | 0.742350 |
| C | 2.421246 | 4.120256 | -1.001354 |
| H | 3.019794 | 4.104822 | -1.923381 |
| H | 1.369388 | 4.096344 | -1.320660 |
| C | 2.693755 | 5.420759 | -0.238457 |
| H | 3.746137 | 5.488976 | 0.063593 |
| H | 2.465209 | 6.299690 | -0.852346 |
| H | 2.083830 | 5.480883 | 0.671480 |
| C | -0.378418 | -1.284580 | -0.668778 |
| C | -0.669584 | -1.759374 | -1.954587 |
| C | -1.453188 | -0.705105 | 0.019097 |
| C | -1.973920 | -1.628860 | -2.482655 |
| H | 0.098355 | -2.233057 | -2.555450 |
| C | -2.732608 | -0.607620 | -0.582141 |
| H | -1.285677 | -0.330657 | 1.019279 |
| H | -2.181788 | -2.005210 | -3.481791 |
| C | -3.857056 | 0.002855 | 0.103830 |
| C | -3.751027 | 0.551384 | 1.421639 |
| C | -6.212938 | 0.644252 | 0.052537 |
| C | -4.846423 | 1.126800 | 2.026986 |
| H | -2.810834 | 0.522020 | 1.955648 |
| C | -6.082890 | 1.178627 | 1.349658 |
| H | -7.169108 | 0.689586 | -0.456646 |
| H | -4.761277 | 1.541642 | 3.024879 |
| H | -6.940492 | 1.633674 | 1.833804 |
| C | 0.965195 | -1.386629 | -0.071414 |
| N | -2.985506 | -1.074442 | -1.838145 |
| C | -5.124863 | 0.066221 | -0.562836 |
| H | -5.194669 | -0.352411 | -1.557689 |

**Table S14.** Cartesian coordinates of the ground state (S_0_) fully optimized geometry in toluene of **CB1B** from B3LYP calculations (in Å)

| Atom | x | y | z |
| --- | --- | --- | --- |
| C | -2.817238 | 0.179451 | 0.204824 |
| H | -4.841544 | -2.916801 | 1.249909 |
| H | -2.534140 | -1.129809 | 2.309221 |
| H | -5.006801 | 0.173571 | 1.363118 |
| H | -6.048642 | -1.395860 | -1.135624 |
| H | -1.874531 | -3.505219 | 0.573865 |
| H | -4.433167 | 1.239468 | -1.375128 |
| H | -4.075204 | -3.703578 | -1.628206 |
| H | -1.600862 | 0.574652 | -1.938095 |
| H | -1.278012 | -2.417506 | -2.134658 |
| H | -3.845594 | -1.109845 | -3.264548 |
| B | -2.894487 | -1.238342 | 1.190730 |
| B | -4.202623 | -2.218967 | 0.531248 |
| B | -4.357135 | -0.445895 | 0.588646 |
| B | -2.517745 | -2.613810 | 0.132314 |
| B | -4.895047 | -1.333370 | -0.855153 |
| B | -4.008217 | 0.192666 | -1.026699 |
| B | -3.754795 | -2.667681 | -1.140772 |
| B | -2.322682 | -0.190696 | -1.403128 |
| B | -2.160037 | -1.962952 | -1.487603 |
| B | -3.621140 | -1.165442 | -2.099107 |
| C | -2.259636 | 1.441845 | 0.873397 |
| H | -2.440356 | 1.360265 | 1.949983 |
| H | -1.172891 | 1.453292 | 0.738928 |
| C | -2.839052 | 2.767903 | 0.360027 |
| H | -3.927473 | 2.775858 | 0.496168 |
| H | -2.654391 | 2.862680 | -0.717360 |
| C | -2.224783 | 3.969600 | 1.091300 |
| H | -2.403410 | 3.868404 | 2.170694 |
| H | -1.134249 | 3.958106 | 0.957158 |
| C | -2.787984 | 5.307565 | 0.602063 |
| H | -3.872573 | 5.360701 | 0.756180 |
| H | -2.332612 | 6.147595 | 1.138199 |
| H | -2.596461 | 5.450172 | -0.468371 |
| C | -0.269172 | -0.970165 | 0.315722 |
| C | 0.186335 | -1.414472 | 1.570503 |
| C | 0.665173 | -0.447059 | -0.578639 |
| C | 1.531031 | -1.298590 | 1.883301 |
| H | -0.485543 | -1.850544 | 2.296694 |
| C | 2.010541 | -0.349788 | -0.205318 |
| H | 0.370719 | -0.113953 | -1.564553 |
| H | 1.925816 | -1.626644 | 2.838102 |
| C | 3.142785 | 0.173734 | -0.962881 |
| C | 4.324395 | 0.086379 | -0.190696 |
| C | 3.121437 | 0.701620 | -2.261625 |
| C | 5.508251 | 0.553760 | -0.773249 |
| C | 4.316630 | 1.157753 | -2.810666 |
| H | 2.199840 | 0.758862 | -2.835511 |
| C | 5.504598 | 1.082905 | -2.066885 |
| H | 6.444342 | 0.508341 | -0.219800 |
| H | 4.329732 | 1.571740 | -3.814959 |
| H | 6.431778 | 1.442663 | -2.506743 |
| C | -1.724615 | -1.112839 | -0.070654 |
| N | 2.413970 | -0.774187 | 1.023188 |
| C | 4.226282 | 0.467890 | 2.508646 |
| H | 3.886588 | 0.037713 | 3.462580 |
| H | 5.293716 | 0.698830 | 2.632372 |
| H | 3.705439 | 1.426659 | 2.377530 |
| C | 4.707811 | -2.019092 | 1.505534 |
| H | 5.798660 | -1.910988 | 1.585182 |
| H | 4.370621 | -2.484463 | 2.443695 |
| H | 4.516186 | -2.737263 | 0.696247 |
| B | 4.024005 | -0.559149 | 1.256961 |

**Table S15.** Cartesian coordinates of the first excited state (S_1_) fully optimized geometry in toluene of **CB1B** from B3LYP calculations (in Å)

| Atom | x | y | z |
| --- | --- | --- | --- |
| C | -3.222629 | 0.195571 | 0.296518 |
| H | -4.542608 | -3.284122 | 1.192333 |
| H | -2.467029 | -1.175699 | 2.238295 |
| H | -5.291591 | -0.421207 | 1.386752 |
| H | -5.914100 | -1.957276 | -1.242003 |
| H | -1.676739 | -3.660756 | 0.587452 |
| H | -4.749180 | 0.892705 | -1.463976 |
| H | -3.706221 | -3.905207 | -1.721317 |
| H | -1.692036 | 0.630957 | -1.626995 |
| H | -1.113023 | -2.319433 | -2.251600 |
| H | -3.663605 | -1.210897 | -3.231967 |
| B | -2.769825 | -1.255732 | 1.089469 |
| B | -3.988134 | -2.487077 | 0.499206 |
| B | -4.487780 | -0.789297 | 0.588135 |
| B | -2.289463 | -2.753879 | 0.118509 |
| B | -4.797216 | -1.708267 | -0.903070 |
| B | -4.178300 | -0.043213 | -1.003565 |
| B | -3.495620 | -2.859263 | -1.186478 |
| B | -2.336095 | -0.222325 | -1.102237 |
| B | -1.969960 | -1.992709 | -1.491346 |
| B | -3.481134 | -1.286301 | -2.055476 |
| C | -2.934818 | 1.499324 | 1.019434 |
| H | -3.421360 | 1.466541 | 2.003095 |
| H | -1.854545 | 1.552019 | 1.221775 |
| C | -3.361332 | 2.778650 | 0.284009 |
| H | -4.447778 | 2.766060 | 0.127169 |
| H | -2.905946 | 2.797358 | -0.715362 |
| C | -2.971649 | 4.052007 | 1.045883 |
| H | -3.423056 | 4.028566 | 2.047920 |
| H | -1.883403 | 4.063458 | 1.201712 |
| C | -3.397840 | 5.334510 | 0.323887 |
| H | -4.485501 | 5.367457 | 0.185056 |
| H | -3.106386 | 6.227166 | 0.889312 |
| H | -2.935468 | 5.401901 | -0.668703 |
| C | -0.091285 | -1.122187 | 0.298824 |
| C | 0.405675 | -1.466434 | 1.572839 |
| C | 0.837135 | -0.541439 | -0.601801 |
| C | 1.734682 | -1.233619 | 1.903875 |
| H | -0.244988 | -1.917520 | 2.311893 |
| C | 2.145857 | -0.332871 | -0.209027 |
| H | 0.522143 | -0.261643 | -1.599501 |
| H | 2.133662 | -1.494314 | 2.877521 |
| C | 3.252524 | 0.245007 | -0.975786 |
| C | 4.452803 | 0.259580 | -0.161571 |
| C | 3.239997 | 0.725604 | -2.273126 |
| C | 5.643928 | 0.781904 | -0.729848 |
| C | 4.437621 | 1.234145 | -2.794760 |
| H | 2.343323 | 0.717596 | -2.882689 |
| C | 5.636616 | 1.260299 | -2.021753 |
| H | 6.555582 | 0.801536 | -0.141383 |
| H | 4.457424 | 1.617098 | -3.809297 |
| H | 6.538496 | 1.662877 | -2.470221 |
| C | -1.481720 | -1.347591 | -0.074380 |
| N | 2.589956 | -0.675990 | 1.035585 |
| C | 4.348433 | 0.754437 | 2.484416 |
| H | 4.045251 | 0.308924 | 3.441065 |
| H | 5.399527 | 1.051984 | 2.582119 |
| H | 3.755025 | 1.663505 | 2.328219 |
| C | 5.009599 | -1.718712 | 1.581414 |
| H | 6.083822 | -1.507701 | 1.647555 |
| H | 4.700199 | -2.139226 | 2.547303 |
| H | 4.864597 | -2.486988 | 0.812322 |
| B | 4.172377 | -0.340515 | 1.273406 |

**Table S16.** Cartesian coordinates of the ground state (S_0_) fully optimized geometry in toluene of **CB2** from B3LYP calculations (in Å)

| Atom | x | y | z |
| --- | --- | --- | --- |
| C | 3.202981 | 0.313320 | 0.006676 |
| H | 5.768179 | -2.517539 | -0.406147 |
| H | 3.742259 | -0.895138 | -2.107343 |
| H | 5.634551 | 0.574667 | -0.389065 |
| H | 5.992118 | -1.027055 | 2.274445 |
| H | 2.821580 | -3.415953 | -0.732721 |
| H | 4.113352 | 1.431271 | 2.043335 |
| H | 4.219513 | -3.531453 | 2.055799 |
| H | 1.333823 | 0.472997 | 1.648781 |
| H | 1.288447 | -2.541568 | 1.668866 |
| H | 3.209442 | -1.065387 | 3.591609 |
| B | 3.735071 | -1.032372 | -0.935351 |
| B | 4.864123 | -1.923341 | 0.085680 |
| B | 4.842137 | -0.144559 | 0.121799 |
| B | 3.192590 | -2.494674 | -0.086892 |
| B | 4.986064 | -1.056324 | 1.641368 |
| B | 3.935274 | 0.369492 | 1.554485 |
| B | 3.961591 | -2.504416 | 1.515103 |
| B | 2.266887 | -0.189305 | 1.358552 |
| B | 2.275096 | -1.970520 | 1.346942 |
| B | 3.372856 | -1.076327 | 2.414605 |
| C | 2.755657 | 1.552396 | -0.778413 |
| H | 3.305806 | 1.566050 | -1.724678 |
| H | 1.697554 | 1.433502 | -1.034721 |
| C | 2.947286 | 2.895256 | -0.059060 |
| H | 4.006292 | 3.037505 | 0.189116 |
| H | 2.398150 | 2.890380 | 0.890898 |
| C | 2.464393 | 4.068926 | -0.922608 |
| H | 3.010027 | 4.066971 | -1.876343 |
| H | 1.404643 | 3.923347 | -1.173822 |
| C | 2.645939 | 5.423869 | -0.231289 |
| H | 3.701130 | 5.611544 | 0.001584 |
| H | 2.293358 | 6.242774 | -0.868180 |
| H | 2.084636 | 5.466373 | 0.709942 |
| C | 0.949865 | -1.060366 | -0.934160 |
| C | 0.949819 | -1.389312 | -2.293980 |
| C | -0.279343 | -0.762699 | -0.338483 |
| C | -0.266104 | -1.384667 | -2.979885 |
| H | 1.859113 | -1.652509 | -2.818279 |
| C | -1.453510 | -0.782913 | -1.109187 |
| H | -0.330300 | -0.524229 | 0.716185 |
| H | -0.286618 | -1.636146 | -4.038259 |
| C | -2.768273 | -0.472989 | -0.533620 |
| C | 2.220313 | -1.089142 | -0.114243 |
| N | -1.440108 | -1.088501 | -2.423206 |
| C | -4.696727 | 0.133922 | 0.971291 |
| C | -5.082724 | -0.117768 | -0.370155 |
| C | -5.628737 | 0.486130 | 1.952304 |
| C | -6.444229 | -0.007813 | -0.717188 |
| C | -6.966392 | 0.587871 | 1.581170 |
| H | -5.319942 | 0.675683 | 2.975895 |
| C | -7.371048 | 0.342520 | 0.254634 |
| H | -6.757495 | -0.197186 | -1.740207 |
| H | -7.707278 | 0.860612 | 2.327172 |
| H | -8.421391 | 0.428724 | -0.008231 |
| S | -2.962443 | -0.057112 | 1.177809 |
| C | -3.962917 | -0.460339 | -1.198061 |
| H | -4.037025 | -0.690434 | -2.254022 |

**Table S17.** Cartesian coordinates of the first excited state (S_1_) fully optimized geometry in toluene of **CB2** from B3LYP calculations (in Å)

| Atom | x | y | z |
| --- | --- | --- | --- |
| C | 3.537705 | 0.467064 | -0.110920 |
| H | 5.610685 | -2.739975 | -0.355710 |
| H | 3.500515 | -1.118091 | -2.031636 |
| H | 5.886618 | 0.197402 | -0.623554 |
| H | 6.097161 | -1.062031 | 2.202484 |
| H | 2.802559 | -3.635736 | -0.385202 |
| H | 4.435130 | 1.515816 | 1.890644 |
| H | 4.226368 | -3.371068 | 2.339497 |
| H | 1.525355 | 0.682289 | 1.353536 |
| H | 1.342570 | -2.309159 | 2.118724 |
| H | 3.335425 | -0.662447 | 3.538200 |
| B | 3.530088 | -1.078907 | -0.840806 |
| B | 4.774319 | -2.031119 | 0.116535 |
| B | 4.982807 | -0.275995 | -0.006778 |
| B | 3.108958 | -2.604489 | 0.127178 |
| B | 5.071134 | -1.046260 | 1.591508 |
| B | 4.164864 | 0.469515 | 1.393192 |
| B | 3.964464 | -2.414496 | 1.674251 |
| B | 2.411657 | -0.060971 | 1.065399 |
| B | 2.281914 | -1.854252 | 1.543358 |
| B | 3.459243 | -0.832253 | 2.362948 |
| C | 3.220610 | 1.662887 | -0.991398 |
| H | 3.894197 | 1.647026 | -1.858656 |
| H | 2.206558 | 1.535540 | -1.399116 |
| C | 3.307694 | 3.037636 | -0.311795 |
| H | 4.330773 | 3.202520 | 0.051223 |
| H | 2.660918 | 3.051667 | 0.576184 |
| C | 2.909465 | 4.183910 | -1.250376 |
| H | 3.551091 | 4.162518 | -2.142903 |
| H | 1.883254 | 4.019507 | -1.609174 |
| C | 3.004104 | 5.561896 | -0.587239 |
| H | 4.027154 | 5.768887 | -0.249622 |
| H | 2.713572 | 6.360090 | -1.280155 |
| H | 2.347513 | 5.624932 | 0.289486 |
| C | 0.762650 | -1.468010 | -0.711208 |
| C | 0.650769 | -2.049592 | -1.987344 |
| C | -0.433796 | -0.971987 | -0.160734 |
| C | -0.589641 | -2.102796 | -2.630486 |
| H | 1.525054 | -2.460276 | -2.479796 |
| C | -1.628093 | -1.070428 | -0.881496 |
| H | -0.417222 | -0.512009 | 0.821433 |
| H | -0.665495 | -2.555714 | -3.616685 |
| C | -2.891737 | -0.564720 | -0.354502 |
| C | 2.040249 | -1.377672 | 0.001578 |
| N | -1.722385 | -1.628610 | -2.112713 |
| C | -4.796178 | 0.433587 | 0.929017 |
| C | -5.193239 | -0.060839 | -0.347335 |
| C | -5.696311 | 1.023959 | 1.790605 |
| C | -6.551367 | 0.056301 | -0.746673 |
| C | -7.040517 | 1.127118 | 1.367289 |
| H | -5.398096 | 1.401363 | 2.762180 |
| C | -7.460571 | 0.650080 | 0.116793 |
| H | -6.859657 | -0.317963 | -1.717018 |
| H | -7.761361 | 1.589346 | 2.033350 |
| H | -8.500786 | 0.747672 | -0.172589 |
| S | -3.063434 | 0.176617 | 1.198924 |
| C | -4.118587 | -0.619169 | -1.056847 |
| H | -4.174700 | -1.056923 | -2.044424 |

**Table S18.** Cartesian coordinates of the ground state (S_0_) fully optimized geometry in toluene of **CB2B** from B3LYP calculations (in Å)

| Atom | x | y | z |
| --- | --- | --- | --- |
| C | -3.645902 | 0.229415 | 0.055611 |
| H | -5.829843 | -2.883700 | 0.612399 |
| H | -3.691208 | -1.204823 | 2.098228 |
| H | -5.980641 | 0.194836 | 0.884225 |
| H | -6.664330 | -1.208656 | -1.826685 |
| H | -2.802138 | -3.487004 | 0.341208 |
| H | -5.004736 | 1.407490 | -1.674999 |
| H | -4.662404 | -3.519407 | -2.166774 |
| H | -2.121527 | 0.721878 | -1.853782 |
| H | -1.812731 | -2.259938 | -2.187084 |
| H | -4.171995 | -0.841268 | -3.596071 |
| B | -3.878062 | -1.241365 | 0.934000 |
| B | -5.086121 | -2.158408 | 0.035581 |
| B | -5.230766 | -0.389870 | 0.176085 |
| B | -3.366384 | -2.561777 | -0.137215 |
| B | -5.562669 | -1.182847 | -1.380474 |
| B | -4.646179 | 0.334351 | -1.332355 |
| B | -4.405945 | -2.519169 | -1.577771 |
| B | -2.926664 | -0.056879 | -1.481262 |
| B | -2.772652 | -1.824980 | -1.646703 |
| B | -4.119727 | -0.966902 | -2.415729 |
| C | -3.176760 | 1.442399 | 0.868596 |
| H | -3.506744 | 1.302795 | 1.902997 |
| H | -2.081735 | 1.444273 | 0.887087 |
| C | -3.667144 | 2.804738 | 0.356693 |
| H | -4.763840 | 2.824154 | 0.342413 |
| H | -3.334758 | 2.954707 | -0.678083 |
| C | -3.147744 | 3.954779 | 1.230615 |
| H | -3.479054 | 3.802451 | 2.267007 |
| H | -2.049404 | 3.928452 | 1.251025 |
| C | -3.618240 | 5.326963 | 0.737930 |
| H | -4.712935 | 5.393886 | 0.737652 |
| H | -3.234279 | 6.129386 | 1.377579 |
| H | -3.272616 | 5.519915 | -0.284902 |
| C | -1.144159 | -0.970644 | 0.448157 |
| C | -0.881398 | -1.362211 | 1.774028 |
| C | -0.075539 | -0.553426 | -0.345904 |
| C | 0.419541 | -1.309948 | 2.251896 |
| H | -1.665546 | -1.712868 | 2.429905 |
| C | 1.216551 | -0.523851 | 0.192579 |
| H | -0.219977 | -0.260746 | -1.377589 |
| H | 0.666425 | -1.602440 | 3.266190 |
| C | 2.469338 | -0.145807 | -0.404114 |
| C | 3.534857 | -0.259978 | 0.457472 |
| C | -2.537041 | -1.065372 | -0.133987 |
| N | 1.438566 | -0.900456 | 1.488264 |
| C | 3.173741 | 0.295673 | 3.106896 |
| H | 2.681381 | -0.046992 | 4.028727 |
| H | 4.233950 | 0.444822 | 3.354023 |
| H | 2.759645 | 1.282805 | 2.860611 |
| C | 3.559556 | -2.280161 | 2.296105 |
| H | 4.638687 | -2.254327 | 2.501307 |
| H | 3.075777 | -2.658982 | 3.208232 |
| H | 3.400672 | -3.025985 | 1.505565 |
| B | 3.031509 | -0.789862 | 1.898822 |
| C | 4.558402 | 0.524253 | -1.548130 |
| C | 4.758122 | 0.126413 | -0.194315 |
| C | 5.619375 | 0.934810 | -2.359576 |
| C | 6.068634 | 0.153942 | 0.324836 |
| C | 6.900817 | 0.949235 | -1.813954 |
| H | 5.453219 | 1.234887 | -3.389920 |
| C | 7.124307 | 0.561175 | -0.479859 |
| H | 6.242429 | -0.145455 | 1.354459 |
| H | 7.738149 | 1.264830 | -2.429958 |
| H | 8.133510 | 0.581266 | -0.078572 |
| S | 2.858190 | 0.423570 | -2.023664 |

**Table S19.** Cartesian coordinates of the first excited state (S_1_) fully optimized geometry in toluene of **CB2B** from B3LYP calculations (in Å)

| Atom | x | y | z |
| --- | --- | --- | --- |
| C | -3.996550 | 0.350554 | 0.197252 |
| H | -5.725156 | -3.058306 | 0.387423 |
| H | -3.575486 | -1.381770 | 1.941017 |
| H | -6.215592 | -0.195161 | 0.991235 |
| H | -6.712376 | -1.224022 | -1.898104 |
| H | -2.861262 | -3.646835 | -0.028445 |
| H | -5.264802 | 1.480634 | -1.544661 |
| H | -4.661639 | -3.303696 | -2.506578 |
| H | -2.249041 | 0.914896 | -1.494255 |
| H | -1.890696 | -1.948656 | -2.546277 |
| H | -4.213196 | -0.428093 | -3.541957 |
| B | -3.766951 | -1.246510 | 0.773524 |
| B | -5.030999 | -2.227964 | -0.114407 |
| B | -5.373743 | -0.519924 | 0.213263 |
| B | -3.334746 | -2.615419 | -0.388585 |
| B | -5.616586 | -1.154393 | -1.430332 |
| B | -4.835069 | 0.426712 | -1.200581 |
| B | -4.408386 | -2.383608 | -1.789883 |
| B | -3.019795 | 0.064937 | -1.175539 |
| B | -2.785135 | -1.652417 | -1.817615 |
| B | -4.157841 | -0.705508 | -2.383084 |
| C | -3.654243 | 1.484801 | 1.146628 |
| H | -4.208224 | 1.334561 | 2.082507 |
| H | -2.589833 | 1.403896 | 1.413269 |
| C | -3.923771 | 2.902219 | 0.619995 |
| H | -4.994809 | 3.016153 | 0.407355 |
| H | -3.402913 | 3.045579 | -0.336401 |
| C | -3.481721 | 3.988252 | 1.609377 |
| H | -3.992951 | 3.834040 | 2.570313 |
| H | -2.407385 | 3.876418 | 1.814355 |
| C | -3.760888 | 5.407738 | 1.104979 |
| H | -4.832434 | 5.560434 | 0.926508 |
| H | -3.432808 | 6.161147 | 1.830407 |
| H | -3.237215 | 5.602691 | 0.160813 |
| C | -1.020398 | -1.279609 | 0.239867 |
| C | -0.685780 | -1.874175 | 1.475818 |
| C | 0.041192 | -0.665295 | -0.469619 |
| C | 0.616565 | -1.842032 | 1.954199 |
| H | -1.446803 | -2.366105 | 2.069127 |
| C | 1.313829 | -0.664351 | 0.067996 |
| H | -0.139408 | -0.197948 | -1.430038 |
| H | 0.888818 | -2.297356 | 2.899477 |
| C | 2.547310 | -0.106653 | -0.456905 |
| C | 3.653179 | -0.319211 | 0.400779 |
| C | -2.381353 | -1.294118 | -0.278332 |
| N | 1.605748 | -1.246210 | 1.271651 |
| C | 3.305119 | -0.254814 | 3.085766 |
| H | 2.841956 | -0.787113 | 3.928262 |
| H | 4.358394 | -0.096666 | 3.353319 |
| H | 2.833401 | 0.734282 | 3.020727 |
| C | 3.834948 | -2.617264 | 1.827265 |
| H | 4.908866 | -2.555843 | 2.048094 |
| H | 3.375372 | -3.181549 | 2.650638 |
| H | 3.723172 | -3.215864 | 0.913988 |
| B | 3.190532 | -1.125420 | 1.712838 |
| C | 4.596396 | 0.875224 | -1.421411 |
| C | 4.830145 | 0.250812 | -0.163554 |
| C | 5.602351 | 1.489597 | -2.140262 |
| C | 6.139724 | 0.264114 | 0.361549 |
| C | 6.901826 | 1.485199 | -1.587514 |
| H | 5.414957 | 1.963351 | -3.097718 |
| C | 7.165181 | 0.881620 | -0.355954 |
| H | 6.338561 | -0.205979 | 1.318791 |
| H | 7.705942 | 1.962812 | -2.137701 |
| H | 8.172900 | 0.893128 | 0.044911 |
| S | 2.880418 | 0.747021 | -1.901091 |
